# Usability of the “Systematic Review Support” computer system: a methodological study *

**DOI:** 10.1590/1518-8345.7081.4374

**Published:** 2024-10-25

**Authors:** Fernanda Martins Dias Escaldelai, Leandro Escaldelai, Denise Pimentel Bergamaschi

**Affiliations:** ^1^ Universidade de São Paulo, Faculdade de Saúde Pública, São Paulo, SP, Brazil.; ^2^ Faculdade de Tecnologia de São Paulo, São Paulo, SP, Brazil.

**Keywords:** Cloud Computing, User-Centered Design, Review, Systematic Review, Software, Information Technology

## Abstract

to evaluate the usability of the “Systematic Review Support” computer system.

methodological study involving 21 students who evaluated the computer system in a remote and synchronous usability test. Two activities were carried out, containing 21 tasks simulating the initial steps of a review study, proposals for eliminating duplicate references, selecting studies and resolving disagreements between evaluators. At the end, participants answered the System Usability Scale questionnaire. The measures used were: completion rate with assistance (effectiveness) and time to complete the task (efficiency). The data was described in a table using the mean, standard deviation, minimum and maximum values.

in a total of 21 tasks, the participants had an assisted completion rate of 9% and an average time of 55.1 minutes (the expected time being 60 minutes). The average score provided by the System Usability Scale instrument was 82.4 (considering a score from 0 to 100), median 85; minimum value 35 and maximum 97.5.

the computer system has the effectiveness and efficiency parameters required of a computer review tool. It has a usability score of 82.4 on a scale of 0 to 100 assessed by the System Usability Scale instrument. The version presented incorporates the adjustments identified in the test in use.

## Highlights

**(1)** The “Systematic Review Support” system has a high degree of perceived usability.

**(2)** Its use facilitates the exclusion of duplicate references and the selection of studies.

**(3)** Remote and synchronous usability testing made research possible during the pandemic.

**(4)** Combining usability techniques made it possible to identify the necessary adjustments.

**(5)** The method used expanded the test’s capacity in a real context of use.

## Introduction

 Review studies that provide an unbiased synthesis of scientific evidence include the selection of studies based on rigorous methods to allow for valid and reproducible results ^(^ 1 ^)^ . The initial stage, which includes searching databases, eliminating duplicate articles and identifying eligible studies, is arduous, especially given the volume of studies to be handled, and the use of computer tools, such as those available on The Systematic Review Toolbox platform, is strongly recommended ^(^ 2 ^)^ . 

 Although Cochrane recommends computer tools for reviews, such as Covidence, EPPI-Reviewer and EndNote® ^(^ 3 ^)^ , there are other products for this purpose ^(^ 2 ^)^ , like Rayyan ^®^ , Parsifal, StArt and the “Systematic Review Support” system (AReS), recently developed to help with the initial steps of reviews in the health area. The limiting factors of computer tools are that access is not always free, the interface is not user-friendly, programming skills are needed and there is a lack of technical support ^(^ 4 ^)^ . AReS overcomes these difficulties and has other advantages, such as a Portuguese-language interface, accurate identification of duplicates without the need for manual work ^(^ 5 ^)^ and support for the selection of eligible studies, presenting the abstract and eligibility criteria registered by the researcher on a single screen ^(^ 6 ^)^ . 

 Recently, there has been a need to draw up syntheses of scientific evidence for the rapid confrontation of the coronavirus disease 2019 (COVID-19) pandemic in the development of new technologies for diagnosis, treatment and control of the disease ^(^ 7 ^)^ . The urgency for up-to-date information has led to innovation and the use of automation to reduce the time it takes to complete the review steps ^(^ 8 ^)^ and for the evaluation of new tools, using the remote usability method to continue research and achieve advances in technology ^(^ 9 ^)^ . 

 There is consensus both in the scientific literature and in standards of good technical quality on the need for usability evaluation of new technological products. Usability refers to the degree to which a product or service can be used by specific users to achieve specified objectives with effectiveness, efficiency and satisfaction in a given context of use ^(^ 2020 ^)^ . 

 The perception of low usability is one of the reasons for not using tools to automate literature reviews ^(^ 11 ^)^ . Usability studies with users carrying out tasks have highlighted that adherence to the use of proofreading software depends on ease of use, quality of the user interface, available resources and functionalities ^(^ 12 ^-^ 14 ^)^ . 

In view of the above, the aim of this study was to evaluate the usability of the “Systematic Review Support” computer system.

## Method

### Type and design of the study

 Methodological study with three steps ^(^ 15 ^-^ 17 ^)^ . In the first step, there were the activities related to the development of the computer system; in the second step, the duplicate reference identification functionality was validated; and in the third, the usability test or in-use test was conducted to identify the usefulness of the tool, the degree of difficulty in use and the adjustments needed to improve the user interface. The in-use test was carried out with volunteers, remotely and synchronously, using the task resolution technique, followed by the application of the System Usability Scale (SUS) questionnaire ^(^ 18 ^-^ 19 ^)^ . 

### Ethical aspects

The project followed the terms of the Resolution of the National Health Council (CNS 466/12) of the Brazilian Ministry of Health and was approved by the Research Ethics Committee of the School of Public Health (FSP) of the University of São Paulo (USP), under Certificate of Presentation for Ethical Appreciation 36397420.8.0000.5421. The Informed Consent Form (ICF) was obtained electronically.

### Systematic Review Support System

 The web system *“Apoio à Revisão Sistemática”* (AReS), available at http://revisaosistematica.com.br , was proposed by the leading author during her PhD course in the Epidemiology Program at FSP/USP, in partnership with an information technology professional, who built it voluntarily. Its functional requirements were based on manuals on systematic reviews; however, the functionalities are generic, catering for other types of review, such as scoping, overview and integrative. 

 In the computer version (desktop or notebook), the system AReS consists of an authentication page that redirects the user to the main page, with a standardized layout for administration sites. In this way, the system allows files to be imported from pre-defined databases such as PubMed, Embase and Web of Science, comparing references to identify duplicates. More than one researcher can take part in the review, either as principal or guest reviewer. AReS is adapted to teamwork, which is common in literature reviews, allowing abstracts to be organized and shared. In the study selection step, the system allows titles and abstracts to be read and, after breaking the blinding process, compares the researchers’ responses on the eligibility of the studies, indicating any discrepancies ^(^ 6 ^)^ . 

 The incremental model used to build the system allows new functionalities to be incorporated in new versions ^(^ 20 ^)^ . The usability evaluation included all the functionalities of the “Abstracts”, “Reviews”, “Groups”, “Criteria” and “Reviewers” menus. 

### Participants and study site

 The usability evaluation took place between March and July 2022, with the participation of students from FSP/USP’s *stricto sensu* postgraduate programs (Professional Master’s in Public Health Entomology; PhD in Epidemiology and Public Health), who were formally attending their courses in 2022. The invitations were sent via email by the FSP/USP Postgraduate Committee. The volunteer sample included postgraduates as they are an audience with a strong interest in conducting reviews and potential users of AReS. The study was restricted to the FSP in order to make it feasible. 

### Eligibility criteria

Inclusion criteria were: having a formal bond to a stricto sensu postgraduate program at FSP/USP; having access to a computer with the Internet and an up-to-date browser; and completing at least 50% of the stage of eliminating duplicate studies using the computer system.

### Sample definition

 The sample size followed that described in the literature, which recommends a minimum sample of eight users performing tasks in a real context of use, in order to obtain reliable results ^(^ 16 ^,^ 21 ^)^ . 

### Tasks and evaluation criteria

**Figure 1:**
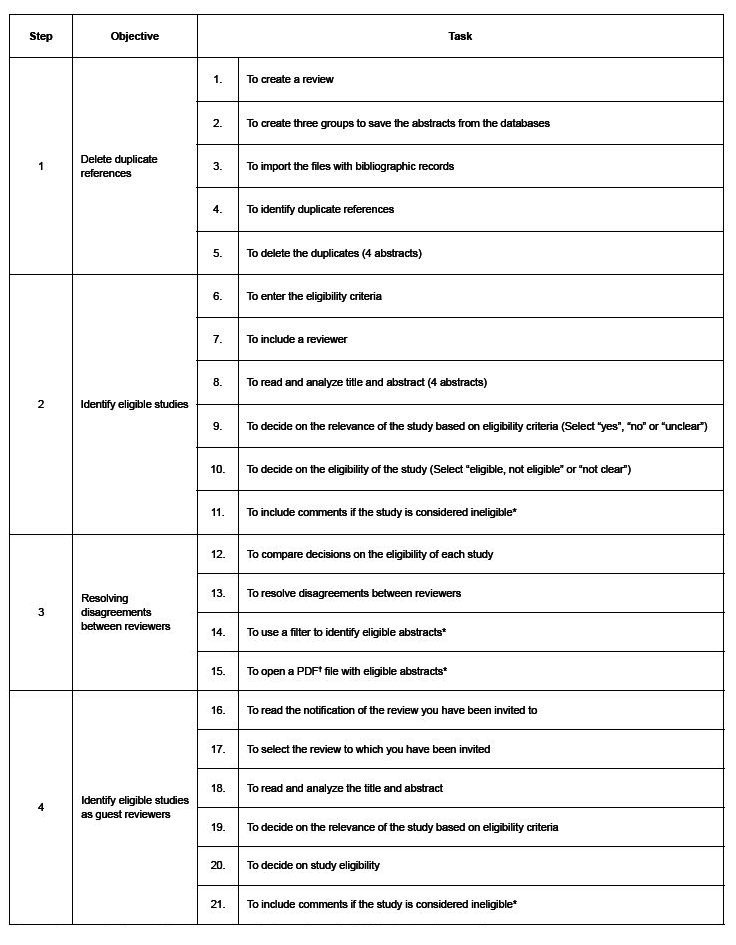
- Intended objective at each stage and tasks to be carried out in the “Systematic Review Support” system

 The evaluation using AReS included two activities, organized into four steps and 21 tasks. In activity 1, steps 1, 2 and 3, each participant took on the role of principal reviewer, responsible for creating a review and organizing abstracts related to the topic of anthropometry in cystic fibrosis. In detail, the tasks involved excluding duplicate references (step 1), blind selection of studies (step 2), breaking the blind, comparing decisions on the eligibility of abstracts and resolving disagreements (step 3). In activity 2, step 4, the participant acted as a guest reviewer in a review organized by the researcher, completing tasks related to identifying eligible studies ( Figure 1 ). 

*Non-mandatory tasks for completing the stage completely and correctly; ^†^ PDF = Portable Document Format

 For each participant, the completeness of the task was recorded, as was the achievement of the expected result and the use of assistance (consultation of explanatory materials on the use of AReS, with videos and tutorials being made available), the occurrence of errors in use resulting from problems with the computer system interfaces, and the time (minutes) taken to complete each task. Error in use was defined as the user’s action (or lack of action) that led to a different result from that intended by the manufacturer or the user ^(^ 16 ^-^ 17 ^)^ . 

The time used by the participant to solve tasks 9, 10, 11, 19, 20 and 21, which informed the decision on the eligibility of the abstracts, was added to the time used to solve tasks 8 and 18, referring to the reading and analysis of titles and abstracts. Completing tasks 5, 8, 13 and 18 was considered mandatory for the definition of completion of the stage, because if the participant did not complete them, it would not be possible to achieve the objectives of excluding duplicate references (stage 1), selecting studies (steps 2 and 4) and resolving disagreements between reviewers (stage 3).

### Data collection

The data was collected individually using a teleconference tool (Google Meet), starting with the provision of information and a link to electronically sign the ICF. The moderator provided a link to download a PDF file (Portable Document Format) with instructions for accessing AReS and the activities to be carried out. There was no prior training on how to use the computer system.

Participants were asked to share the screen of their device in order to record and later check whether they had completed the tasks, whether they needed assistance to solve them and whether there were any errors when using the computer system. The information was recorded by the researcher on a form. The expected time to complete all the tasks was 60 minutes. The time of the actions and clicks made in AReS were recorded in a log file, saved in an internal database.

During the session, questions were answered about the wording of the tasks, but not about how to solve them. The instructions for use contained in AReS could be consulted at any time. When the participant verbalized that there was difficulty in completing a task, they were encouraged to continue trying to solve it, and assistance was offered only when the problem could not be solved. The assistance consisted of a link to a website containing explanatory material on how to use the computer system.

 After completing the tasks, the participants completed two electronic questionnaires: one on the characterization of the participants and the other containing the Portuguese-language version of the SUS instrument ^(^ 19 ^)^ to evaluate the usability of AReS. Comments on its use and suggestions for improvements were requested. 

### Data analysis

 For the in-use evaluation, the task was completed with or without assistance (effectiveness), the resolution time (efficiency) and the degree of usability (SUS instrument), as recommended in technical standards ^(^ 16 ^-^ 17 ^)^ . 

 For effectiveness, the following metrics were chosen: ( *i* ) task completion rate (percentage of participants who achieved the objective of each task completely and correctly), ( *ii* ) assisted completion rate (percentage of participants who completed each task correctly, consulting the explanatory materials), ( *iii* ) system usage error rate (percentage of tasks with results different from those intended due to problems with the interface) ^(^ 16 ^-^ 17 ^)^ . 

Unexpected actions, identified through non-intrusive observation, were noted down and made it possible to identify the errors in use and indicate the necessary adjustments to the computer system. The absolute and relative frequencies of the results related to their effectiveness, errors in use and the type of explanatory material consulted were described.

 With regard to efficiency, a quality property that indicates the resources used to complete the tasks, it was decided to measure the time spent solving them as a proxy for the degree of difficulty encountered and the consequent amount of resources used, as guided by the technical standard ^(^ 16 ^-^ 17 ^)^ . Measuring time in use is a proxy for measuring the resources involved because it would be necessary to measure the human effort required for the computer system to produce the expected results. This effort could be indicated by the number of clicks, use of functionalities and menus, which would be a complex task in this research as it involves quantifying all the actions recorded by the computer system in a text file. 

The efficiency results are presented in a table, using the mean, standard deviation, minimum and maximum values, as well as the 95% Confidence Interval (95%CI) for the mean, according to stage.

 The usability construct, measured using the SUS instrument ^(^ 19 ^)^ , allowed the usability score to be obtained. This instrument contains 10 items graded using a Likert-type scale, with values from 1 to 5 corresponding respectively to “strongly disagree”, “partially disagree”, “neither agree nor disagree”, “partially agree” and “strongly agree”. In the final score, for the odd-numbered questions, the score was calculated as the mark received minus 1, while for the even-numbered questions, it was calculated as 5 minus the mark received. The odd and even scores were added together and multiplied by 2.5 to obtain the final score. The final scores were presented in usability categories: worst imaginable (score from 0 to 24.9); poor (from 25.0 to 38.9); average (from 39.0 to 51.9); good (from 52.0 to 73.9); excellent (from 74.0 to 84.9); and best imaginable (from 85.0 to 100) ^(^ 22 ^)^ . 

## Results

### Characterization of the participants

 A total of 21 students took part, with an average age of 39 years (standard deviation=7 years; minimum=28 and maximum=59 years). Twelve (57%) were female, 20 (95%) were doctoral students and one (5%) was a master’s student. Eighteen (86%) had previously carried out some kind of review study, whether systematic (n=9; 53%), narrative (n=5; 29%) or both (n=3; 18%). Six (33%) reported having used some kind of software to carry out a review, with EndNote ^®^ being the most frequently used, followed by Excel ^®^ , Rayyan ^®^ , Mendeley ^®^ and Word ^®^ . Individuals who signed the electronic informed consent form but did not start the activities on the computer system or did not respond to the research instruments were excluded (n=2). 

### Effectiveness

 In the evaluation process, a completion rate of 90% (n=19 tasks) was obtained in steps 1 and 3 (referring to the exclusion of duplicates and resolution of disagreements) and 95% (n=20) in steps 2 and 4 (selection of studies). In tasks 14 and 21, the completion rates were lower than 90%; however, there was no interference in achieving the objective of each stage. Among the tasks completed, those with the highest percentage of assistance were 12 (comparing decisions on study eligibility) and 17 (selecting the review in which they were invited). The percentage of assistance among those who completed the tasks was 9%. Checking which tasks had the lowest completion rates allowed us to identify some of the adjustments to be made to the computer system ( Table 1 ). 

 For the completed tasks, there were 34 moments of assistance, with access to explanatory videos at 28 moments: tasks 6 (n=1); 8 (n=3); 12 (n=12); 16 (n=3); 17 (n=8) and 18 (n=1), and reading tutorials at six moments: tasks 4 (n=1); 12 (n=1); 13 (n=1); 14 (n=1) and 17 (n=2). The materials were chosen by the participant based on the technological resources available, such as internet quality, at the time of the test. The videos were also used to assist with three uncompleted tasks (4, 8, 12). This data is not shown in Table 1 , which refers only to the completed tasks. 

**Table 1:** - Distribution of participants who solved the tasks using the “Systematic Review Support” system, according to the measures of task completion, assistance and error in use. São Paulo, SP, Brazil, 2022

Step*	**Task**		**Completion rate**		**Conclusion with** **assistance**			**Error in use**	
			n	%	Total	n	%	n	%
1	1.	To create a review	21	100	21	0	-	6	29
	2.	To create three groups to save the abstracts from the databases	21	100	21	0	-	0	-
	3.	To import the files with bibliographic records	21	100	21	0	-	0	-
	4.	To identify duplicate references	19	90	19	1	5	0	-
	5.	To delete the duplicates (4 abstracts)	19	90	19	0	-	0	-
2	6.	To enter the eligibility criteria	21	100	21	1	5	0	-
	7.	To include a reviewer	21	100	21	0	-	0	-
	8.	To read and analyze titles and abstracts (4 abstracts)	20	95	20	3	15	16	80
	9.	To decide on the relevance of the study based on the eligibility criteria.	21	100	21	0	-	0	-
	10	To decide on the eligibility of the study	21	100	21	0	-	0	-
	11.	To include comments if necessary ^†^	21	100	21	0	-	0	-
3	12.	To compare the eligibility decisions of each study	19	90	19	13	68	5	26
	13	To resolve disagreements between reviewers	19	90	19	1	5	0	-
	14	To use a filter to identify eligible abstracts ^†^	12	57	12	1	8	0	-
	15	To open a PDF ^‡^ file with eligible abstracts ^†^	19	90	19	0	-	0	-
4	16.	To read the notification of the review in which you have been invited	21	100	21	3	15	11	52
	17.	To select the review to which you have been invited	20	95	20	10	55	0	-
	18.	To read and analyze the title and abstract	20	95	20	1	5	8	40
	19.	To decide on the relevance of the study based on eligibility criteria	20	95	20	0	-	0	-
	20.	To decide on study eligibility	20	95	20	0	-	0	-
	21.	To include comments if necessary†	15	71	15	0	-	0	-
**Total**			**411**	**93**	**411**	**34**	**9**	**46**	**11**

* Step: 1) Remove duplicates; 2) Identify eligible studies; 3) Resolve disagreements between reviewers; 4) Identify eligible studies as a guest reviewer

^†^ Non-mandatory tasks to complete the stage fully and correctly

^‡^ PDF = Portable Document Format

**Figure 2:**
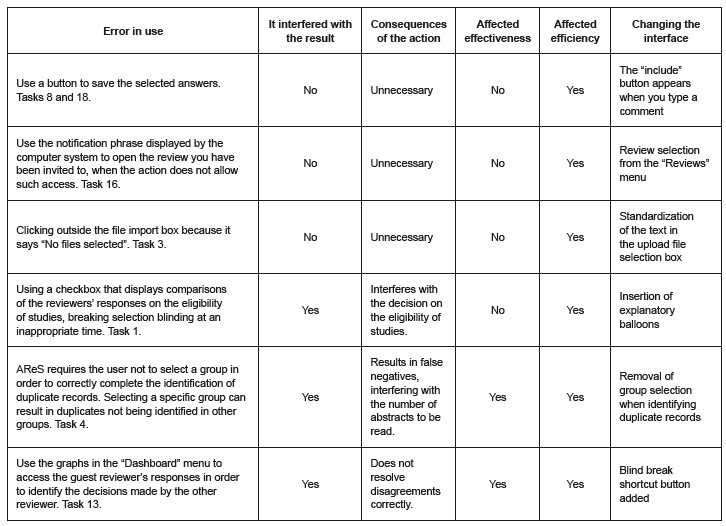
- Actions taken by the participants that interfered with the quality properties of the “Systematic Review Support” system or problems in the computer system interface that did not allow the desired result.

 During resolution, usage errors emerged for both completed ( Table 1 ) and uncompleted tasks, such as 4 (n=4), 8 (n=1), 12 (n=1) and 13 (n=1). These occurrences made it possible to identify the adjustments needed in AReS, depending on the type of error and the consequence of its occurrence, which may (or may not) interfere with its effectiveness or efficiency. Details of the errors in use observed and the results of the analysis are shown in Figure 2 . 

### Efficiency

 Using AReS required an average total time of 55.1 minutes (standard deviation=15.8). To solve the tasks, according to the objective sought at each stage, the average time ranged from 12.4 to 15.8 minutes ( Table 2 ). 

**Table 2:** - Average time (minutes) to complete the steps using the “Systematic Review Support” system (n = 21). São Paulo, SP, Brazil, 2022

	**Step/objective**	**Average time** **(minutes)**	SD*	**Median**	**Minimum** **maximum**	**(95%)CI** ^†^
1	Exclusion of duplicate records	12.8	7.0	11.7	4-31	10-16
2	Identification of eligible studies	15.8	6.8	14.9	7-32	13-19
3	Resolving disagreements	14.0	8.1	12.8	1-32	10-18
4	Selection of eligible studies, as invited	12.4	4.2	12.9	4-20	11-14
Total evaluation process		55.1	15.8	55.1		

* SD = Standard deviation

^†^ (95%)CI= 95% Confidence interval

### AReS usability

**Figure 3:**
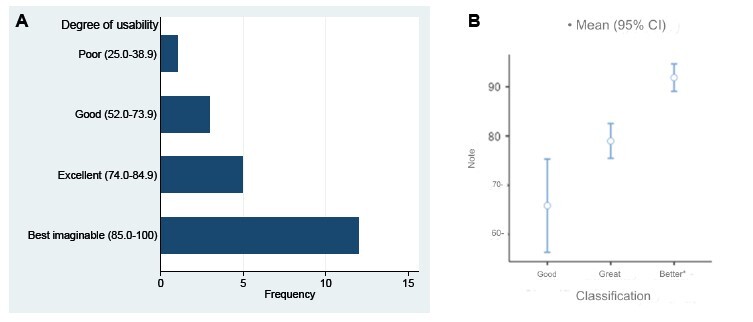
- Distribution of participants according to score (A) and average score per usability category (B) of the System Usability Scale instrument for the “Systematic Review Support” system

 The final average usability score for AReS was 82.4; median 85; minimum and maximum values 35 and 97.5 (on a scale of 0 to 100). The scores for this construct per participant were: “best imaginable” (n=12; 57%), “excellent” (n=5; 24%), “good” (n=3; 14%) and “poor” (n=1; 5%). Figure 3 shows the frequencies, average final scores and (95%)CI according to the categories of usability when using the computer system. It should be noted that the mean and (95%)CI for the “poor” category are not shown, as it has one participant. Using the (95%)CI, it can be said that the “best imaginable” category stands out when compared to the “good” and “excellent” categories, the latter being the category with the highest representation of participants ( Figure 3 ). 

*(95%)CI = 95% Confidence interval

#### General conditions observed during the AReS use test

The most used operating system was Microsoft Windows with the Chrome browser (n=18), followed by MacOS with the Safari browser (n=2). Errors were observed in Safari, such as non-importation of files and automatic selection of decisions on study eligibility, showing the need for adjustments to AReS. During the test, participants switched to the Chrome browser to make better use of the computer system and to complete tasks 3 and 8.

The camera was kept open by the majority of participants (n=14). One said he would keep it closed due to problems with its operation. The participant was asked to “think out loud”, and nine (43%) followed this instruction. Most participants shared the entire screen, however, when only the AReS usage tab was shared, the researcher was unable to check the type of explanatory material used.

Rescheduling the test was necessary for two participants due to slow internet speeds and problems with their computer. The AReS system presented problems when pressing the icon to select the review in which the participant was invited (Task 17), making it necessary to press the refresh icon or log in again. In activity 2 (invited reviewer), the computer system was slow and had technical problems which prevented some participants from completing Task 21.

Some participants made suggestions: to use Portuguese-language summaries in the next test (n=3) and to make it clear at the beginning that participation would be as the main reviewer and then as a guest (n=1). One participant complained about the large number of summaries that had to be read (n=8 abstracts) and another said that the PDF with the tasks didn’t explain how to solve them, even after the researcher told them that they should read the task and try to solve it on their own in the computer system.

#### Comments from participants

The participants’ suggestions on AReS are presented below:

Improve the database import box (...) to induce selection of the import file (P1).

Include a guidance icon on attention to detail on the screen and explanation of each item (P2).

I found the system very intuitive. However, I couldn’t identify how to change eligibility when there is a discrepancy between authors. The graph on the dashboard only showed me the percentage of eligible studies for each author, giving the false impression that there were no discrepancies. But when I looked again, there were discrepancies (P3).

I think the user interface is difficult and unintuitive, it’s hard to figure out the next step. I think there is a lack of information boxes on how to operate the system or how to perform a certain task. I don’t know what it would be like if I’d had prior training on the system, what it would be like to use it, but as the tests were without full training (just some handout data), I found it quite difficult (P4).

### Adjustments and improvements

Adjustments and improvements included: standardizing, in all browsers, the text of the file selection box for uploading; including explanatory balloons over the buttons on the computer system screens; including a button for breaking blinding, to make it easier to display the comparison of reviewers’ responses; allowing the selection of a review via the “Reviews” menu (and not just via the top bar); list separately the reviews created by the researcher himself and the reviews in which he was invited to participate; on the abstracts screen, display the save button only when a comment is included; allow clicking on the title of the study to access the abstract; when identifying duplicates, remove the option to select a group for comparing references.

## Discussion

 This study evaluated the “Systematic Review Support” (AReS) system, which provides functionalities for the initial steps of literature review studies, structured according to a careful methodology ^(^ 1 ^,^ 3 ^)^ , also following the requirements of international standards ^(^ 16 ^)^ . The empirical evaluation showed a high degree of perceived usability on the part of the volunteer evaluators, who were graduate students and who, unintentionally, constituted a heterogeneous group in terms of the use of computer tools in review studies, being representative of the target audience. 

 The possibility of selection bias should always be considered when using volunteer sampling, since the motivation for using the software may have been due to an interest in getting to know the new tool or the opportunity to learn how to use it ^(^ 22 ^)^ . The second option is corroborated by the 67% of participants who reported not having used this type of tool before. This characteristic may have had a positive effect on the study, since they could have needed more computer resources and demanded more from the system being evaluated ^(^ 23 ^)^ . 

The methodological option of not having prior training allowed for exposure to a new product and favored the emergence of information regarding the difficulties of use due to the computer system’s interfaces, giving the test genuineness and fulfillment of the study’s objective.

 The tasks proposed in the test in use were created with a step-by-step approach in mind ^(^ 24 ^)^ for carrying out a review, following a four-stage sequence, which was conducive to verifying effectiveness, efficiency and monitoring interface problems. As the quality of a review depends on the unbiased selection of studies ^(^ 3 ^)^ , the strategy of more than one reviewer was contemplated in AReS, resulting in a longer evaluation, carried out in two steps, each with different roles for the reviewer (main and guest). 

The participants achieved the objectives of the step in high percentages, with a completion rate of at least 90%, considered satisfactory by the authors, giving the evaluation process the quality of internal validity, indicating that the methodological resources used were able to capture the adequacy of the AReS functionalities.

For tasks 14 (to use of a filter to identify eligible abstracts) and 21 (inclusion of comments if the study is considered ineligible by the guest reviewer), which had completion rates of less than 90%, the influence on the software’s performance is minimal, indicating through task 14 a possible problem in the specification of the task, rather than in the interface. With the second occurrence, the need to correct technical problems in the system can be identified. Only task 12 was considered complex, as it involved accessing more than one menu to reach its conclusion, justifying the assistance required by the majority of participants and indicating the need to include a shortcut button.

 The average usability test time varied as expected by the researchers. When conducting a search, the average time taken to read and analyze titles and abstracts (task 8) varies according to the number of references retrieved from the databases, while the time taken to resolve discrepancies can vary according to the number of abstracts to be compared ^(^ 25 ^)^ . The findings of this study corroborate the literature, which indicates that the initial steps of reviews require the most time to complete the selection of eligible studies, so the preference is for resources that favors a reduction in the time dedicated to this stage ^(^ 13 ^)^ , in relation to manual work and non-specific tools such as spreadsheets. 

 AReS was rated positively in terms of usability, with “best imaginable” in 57% of the evaluations; 24% as “excellent” and 14% as “good”. The SUS questionnaire was easy to apply and is a validated instrument that has been widely used in research and tests conducted by information technology professionals, including in the health sector ^(^ 26 ^)^ . It should be noted that the SUS tool also allows comparisons to be made between different web systems ^(^ 27 ^)^ , with users in different geographical regions ^(^ 28 ^)^ . 

 The occurrence of errors in use, such as the failure to identify all duplicates, generally indicated the need for greater communication between the computer system and the user, resolved by adjustments to the interface and the inclusion of explanatory messages. Identifying problems in use and correcting them makes the product more intuitive to use and more popular with the target audience ^(^ 29 ^)^ . 

Remote testing has already been described in the literature and was important because it allowed participants to use their technological resources, enabling usability testing in a real context of use. It also allowed the recording of the work session and the subsequent recording of data, such as the identification of completeness, the fulfillment of the task objective and the occurrence of errors in use. The use of a teleconferencing tool was fundamental and allowed interaction between those involved, facilitated the sharing of instructional materials for the activities, the observation and recording of screens, without the need for additional costs for printing materials and filming equipment.

 Although there are differences in intra-municipal connectivity ^(^ 30 ^)^ , the conditions under which the test was conducted were considered adequate by the researchers. According to the literature, remote synchronous testing does not differ significantly from laboratory testing ^(^ 31 ^)^ and favors the participation of people located in different regions of the country or abroad, and may be preferred in research with a low budget for this type of evaluation and specific demands, such as the social isolation imposed by the COVID-19 pandemic. 

As a potentiality of this study, it was mandatory to carry out the usability test in accordance with recognized methodological procedures, in order to have reliable, effective software with a high degree of usability. Technologies that produce accurate results are crucial to aid decision-making and guarantee data security and accuracy. Including the product’s evaluation process in detail in this work can help health professionals who take part in other evaluation processes, as it presents specific tools for evaluating software, provides a basis for the choices of use and explains the quality properties to be evaluated.

 The important characteristics for choosing specific computer programs in a systematic review are simplicity, ease of learning and intuitive layout ^(^ 12 ^)^ . The construction of the product presented sought to achieve these attributes. In order to control bias in the selection of eligible studies, AReS incorporated the possibility of more than one evaluator participating and the blinding of decisions, guaranteeing the independence of their decisions. Another feature of AReS is the presentation, on a single screen, of the abstract of the article and the eligibility criteria which, when met, lead to the decision to be eligible (or not). This functionality has not been found in any other system and is an original and unique proposal for AReS. 

The study’s limitations refer to the reason for building AReS, which originated from the researcher’s specific need to operationalize a review. It is presented only in Portuguese, including the support materials. The instrument used to evaluate usability (SUS) has been validated for Portuguese in Portugal; in planning the test, no detailed cross-cultural adaptation study of this instrument for the Brazilian context was retrieved.

Also, as a methodological limitation, we can mention the inhomogeneity of the procedure for opening the cameras during the test in use. However, this fact does not seem to have interfered with the results, since it was not of interest to evaluate the behavioral dimension of using the tool. The failure to share the screen during the use of explanatory material was also a limiting factor. It should be noted, nevertheless, that the researcher asked for this information to be recorded on the form. The failure to clarify to a participant the role they would play as the main or guest reviewer can be considered a methodological flaw in the test. This occurrence was verbalized by the second participant and guidance was given to all the others.

 In relation to other computer tools, the current version of AReS has functionalities for eliminating duplicates and selecting studies, unlike those that support other steps of a systematized review, such as data extraction, aided by DistillerSR ^(^ 32 ^)^ . AReS does not highlight keywords, nor does it allow full articles to be imported, as Rayyan does. 

 As a vision for the future, we can highlight the development of functionalities for other review steps, using artificial intelligence applications, as well as other available computational tools ^(^ 33 ^-^ 34 ^)^ , could increase its use by students, researchers and health professionals. 

## Conclusion

AReS has the effectiveness and efficiency parameters required of a computerized review tool. It has a usability score of 82.4 on a scale of 0 to 100 assessed by the System Usability Scale (SUS) instrument. The version presented incorporates the adjustments identified in the test in use. AReS is a useful and easy-to-use tool, aimed at the academic and research environment, proposed to reduce work time in the initial steps of literature reviews.

## References

[B1] Aromataris E., Munn Z. (2020). JBI Manual for Evidence Synthesis [Internet]. JBI.

[B2] Johnson E. E., O’Keefe H., Sutton A., Marshall C. (2022). The Systematic Review Toolbox: keeping up to date with tools to support evidence synthesis. Syst Rev.

[B3] Lefebvre C., Glanville J., Briscoe S., Featherstone R., Littlewood A., Metzendorf M. I., Higgins J. P. T., Thomas J., Chandler J., Cumpston M., Li T., Page M. J. (2023). Chapter 4: Searching for and selecting studies. Cochrane Handbook for Systematic Reviews of Interventions version.

[B4] Kohl C., McIntosh E. J., Unger S., Haddaway N. R., Kecke S., Schiemann J. (2018). Online tools supporting the conduct and reporting of systematic reviews and systematic maps: a case study on CADIMA and review of existing tools. Environ Evid.

[B5] Escaldelai F. M. D., Escaldelai L., Bergamaschi D. P. (2023). Avaliação de validade de um sistema computacional na identificação de estudos duplicados. Esc Anna Nery.

[B6] Escaldelai F. M. D., Escaldelai L., Bergamaschi D. P. (2022). Sistema "Apoio à Revisão Sistemática": solução web para gerenciamento de duplicatas e seleção de artigos elegíveis. Rev Bras Epidemiol.

[B7] Leineweber F. V., Bermudez J. A. Z. (2021). Technologies for COVID-19 and innovative therapies: contemporary challenges. Cad Saude Publica.

[B8] Knottnerus J. A., Tugwell P. (2020). Methodological challenges in studying the COVID-19 pandemic crisis. J Clin Epidemiol.

[B9] Sherwin L. B., Yevu-Johnson J., Matteson-Kome M., Bechtold M., Reeder B. (2022). Remote Usability Testing to Facilitate the Continuation of Research. Stud Health Technol Inform.

[B10] Weichbroth P. (2020). Usability of mobile applications: a systematic literature study. IEEE Access.

[B11] AJ Altena, R Spijker, SD Olabarriaga (2019). Usage of automation tools in systematic reviews. Res Synth Methods.

[B12] Cleo G., Scott A. M., Islam F., Julien B., Beller E. (2019). Usability and acceptability of four systematic review automation software packages: a mixed method design. Syst Rev.

[B13] Harrison H., Griffin S. J., Kuhn I., Usher-Smith J. A. (2020). Software tools to support title and abstract screening for systematic reviews in healthcare: an evaluation. BMC Med Res Methodol.

[B14] Polit D. F., Beck C. T. (2019). Fundamentos de pesquisa em enfermagem: avaliação de evidências para a prática da enfermagem.

[B15] Associação Brasileira de Normas Técnicas (2011). ABNT NBR ISO/IEC 25062: Software engineering: requirements and evaluation of software product quality (SQuaRE) – Common industry format (FCI) for usability testing reports [Internet].

[B16] Associação Brasileira de Normas Técnicas (2021). ABNT NBR ISO 9241-11: Ergonomia da interação humano-sistema. Parte 11: Usabilidade: Definições e conceitos [Internet].

[B17] Brooke J., Jordan P. W., Thomas B., Weerdmeester B. A. (1996). Usability evaluation in industry [Internet].

[B18] Martins A. I., Rosa A. F., Queirós A., Silva A., Rocha N. P. (2015). European Portuguese validation of the System Usability Scale (SUS). Procedia Comp Sci.

[B19] Sommerville I. (2018). Engenharia de Software.

[B20] British Standards Institution (2012). BSI ISO/IEC 25041. Systems and software engineering - Systems and software Quality Requirements and Evaluation (SQuaRE) - Evaluation guide for developers, acquirers and independent evaluators [Internet].

[B21] Bangor A., Kortum P., Miller J. (2009). Determining what individual SUS scores mean: adding an adjective rating scale. J Usability Stud [Internet].

[B22] Pereira M. G. (2021). Epidemiologia: teoria e prática.

[B23] Hill J., Brown J., Campbell N., Holden R. (2021). Usability-In-Place-Remote Usability Testing Methods for Homebound Older Adults: Rapid Literature Review. JMIR Form Res.

[B24] Muka T., Glisic M., Milic J., Verhoog S., Bohlius J., Bramer W. (2020). A 24-step guide on how to design, conduct, and successfully publish a systematic review and meta-analysis in medical research. Eur J Epidemiol.

[B25] Nussbaumer-Streit B., Ellen M., Klerings I., Sfetcu R., Riva N., Mahmić-Kaknjo M. (2021). Resource use during systematic review production varies widely: a scoping review. J Clin Epidemiol.

[B26] Almeida A. F., Rocha N. P., Silva A. G. (2020). Methodological quality of manuscripts reporting on the usability of mobile applications for pain assessment and management: a systematic review. Int J Environ Res Public Health.

[B27] Kortum P., Acemyan C. Z., Oswald F. L. (2020). Is It Time to Go Positive? Assessing the Positively Worded System Usability Scale (SUS). Hum Factors.

[B28] Kortum P., Acemyan C. Z. (2019). The Impact of Geographic Location on the Subjective Assessment of System Usability. Int J Hum Comput Interact.

[B29] JongWook J., NeungHoe K., Hoh P. I. (2020). Detecting usability problems in mobile applications on the basis of dissimilarity in user behavior. Int J Hum Comput Stud.

[B30] Núcleo de Informação e Coordenação do Ponto BR (2022). ICT Households: Survey on the use of information and communication technologies in Brazilian households 2021 [Internet]. Brazilian Internet Steering Committee.

[B31] Sauer J., Sonderegger A., Heyden K., Biller J., Klotz J., Uebelbacher A. (2019). Extra-laboratorial usability tests: an empirical comparison of remote and classical field testing with lab testing. Appl Ergon.

[B32] Khalil H., Ameen D., Zarnegar A. (2022). Tools to support the automation of systematic reviews: a scoping review. J Clin Epidemiol.

[B33] R Cierco Jimenez, T Lee, N Rosillo, R Cordova, IA Cree, A Gonzalez (2022). Machine learning computational tools to assist the performance of systematic reviews: A mapping review. BMC Med Res Methodol.

[B34] Santos A. O. D., Silva E. S., Couto L. M., Reis G. V. L., Belo V. S. (2023). The use of artificial intelligence for automating or semi-automating biomedical literature analyses: A scoping review. J Biomed Inform.

