# Supplementary material for: Usability of the “Systematic Review Support” computer system: a methodological study
Source: Rev Lat Am Enfermagem. 2024 Oct 25;32:e4374. doi: 10.1590/1518-8345.7081.4374 (PMC11526212; doi:10.1590/1518-8345.7081.4374)
Supplement: Translation

# Translation: Usabilidad del sistema computacional “Apoyo a la Revisión Sistemática”: un estudio metodológico*

**DOI:** 10.1590/1518-8345.7081.4373

**Published:** 2024-10-07

**Authors:** Fernanda Martins Dias Escaldelai, Leandro Escaldelai, Denise Pimentel Bergamaschi

**Affiliations:** ^1^ Universidade de São Paulo, Faculdade de Saúde Pública, São Paulo, SP, Brasil.; ^2^ Faculdade de Tecnologia de São Paulo, São Paulo, SP, Brasil.

**Keywords:** Nube Computacional, Diseño Centrado en el Usuario, Revisión, Revisión Sistemática, Programas Informáticos, Tecnología de la Información, Prueba de Usabilidad

## Abstract

evaluar la usabilidad del sistema computacional “Apoyo a la Revisión Sistemática”

estudio metodológico involucrando a 21 estudiantes que evaluaron el sistema computacional en un test de usabilidad remoto y sincrónico. Se realizaron dos actividades, conteniendo 21 tareas simulando las etapas iniciales de un estudio de revisión, propuestas para la eliminación de referencias duplicadas, selección de estudios y resolución de divergencias entre evaluadores. Al final, los participantes respondieron al cuestionario *System Usability Scale* . Las medidas utilizadas fueron: tasa de conclusión con soporte (eficacia) y tiempo para concluir la tarea (eficiencia). Los datos fueron descritos en tabla mediante media, desviación estándar, valores mínimo y máximo.

en el total de 21 tareas, los participantes presentaron una tasa de conclusión con soporte del 9% y un tiempo medio de 55,1 minutos (siendo el tiempo esperado: 60 minutos). La nota media proporcionada por el instrumento *System Usability Scale* fue de 82,4 (considerando una puntuación de 0 a 100), mediana 85; valor mínimo 35 y máximo 97,5.

el sistema computacional presenta parámetros de eficacia y eficiencia necesarios para una herramienta computacional de revisión. Presenta usabilidad de 82,4 en escala de 0 a 100 evaluada por el instrumento *System Usability Scale* . La versión presentada incorpora los ajustes identificados en el test en uso.

## Destacados

**(1)** El sistema “Apoyo a la Revisión Sistemática” posee un alto grado de usabilidad percibida.

**(2)** Su uso facilita la eliminación de referencias duplicadas y la selección de estudios.

**(3)** El test de usabilidad remoto y sincrónico hizo viable la investigación durante la pandemia.

**(4)** Asociar técnicas de usabilidad permitió identificar los ajustes necesarios.

**(5)** El método utilizado amplió la capacidad del test en contexto real de uso.

## Introducción

 Los estudios de revisión que proporcionan una síntesis imparcial de las evidencias científicas incluyen la selección de estudios basada en métodos rigurosos para permitir resultados válidos y reproducibles ^(^ 1 ^)^ . La etapa inicial, que incluye búsquedas en bases de datos, eliminación de artículos duplicados e identificación de estudios elegibles, es ardua, especialmente debido al volumen de estudios a manejar, siendo altamente recomendada la utilización de herramientas computacionales, como las disponibles en la plataforma *The Systematic Review Toolbox* ^(^ 2 ^)^ . 

 A pesar de que *Cochrane* recomienda herramientas computacionales para revisiones, como *Covidence* , *EPPI-Reviewer* y *EndNote* ^®(3)^ , existen otros productos con ese propósito ^(^ 2 ^)^ , como *Rayyan* ^®^ , *Parsifal* , *StArt* y el sistema “Apoyo a la Revisión Sistemática” (AReS), desarrollado recientemente para dar soporte en las etapas iniciales de revisiones en el área de la salud. Se pueden encontrar, como limitadores de herramientas computacionales, el acceso no siempre gratuito, la interfaz no amigable, la necesidad de conocimientos de programación y la falta de soporte técnico ^(^ 4 ^)^ . El AReS supera tales dificultades y presenta otras ventajas, como interfaz en lengua portuguesa, identificación precisa de duplicados sin necesidad del trabajo manual ^(^ 5 ^)^ y apoyo a la selección de estudios elegibles, presentando en una sola pantalla el resumen y los criterios de elegibilidad registrados por el investigador ^(^ 6 ^)^ . 

 Recientemente, hubo la necesidad de elaborar síntesis de evidencias científicas para el enfrentamiento rápido de la pandemia de *coronavirus* 2019 (COVID-19) en el desarrollo de nuevas tecnologías para diagnóstico, tratamiento y control de la enfermedad ^(^ 7 ^)^ . La urgencia por información actualizada indujo a la innovación y al uso de la automatización para reducir el tiempo de conclusión de las etapas de revisión ^(^ 8 ^)^ y para la evaluación de las nuevas herramientas, con uso del método de usabilidad remoto para la continuidad de las investigaciones y la obtención de avances en tecnología ^(^ 9 ^)^ . 

 Existe consenso tanto en la literatura científica como en normas de buena calidad técnica sobre la necesidad de evaluación de la usabilidad de nuevos productos tecnológicos. La usabilidad se refiere al grado en que un producto o servicio puede ser utilizado por usuarios específicos para alcanzar los objetivos especificados con eficacia, eficiencia y satisfacción en un determinado contexto de uso ^(^ 2020 ^)^ . 

 La percepción de usabilidad calificada como baja es uno de los motivos para desistir de la utilización de herramientas para la automatización de revisiones de literatura ^(^ 11 ^)^ . Estudios de usabilidad con realización de tareas por los usuarios destacaron que la adhesión al uso de *software* de revisión depende de la facilidad de utilización, calidad de la interfaz de usuario, recursos y funcionalidades disponibles ^(^ 12 ^-^ 14 ^)^ . 

Considerando lo expuesto, el objetivo de este estudio fue evaluar la usabilidad del sistema computacional “Apoyo a la Revisión Sistemática”.

## Método

### Tipo y diseño del estudio

 Estudio metodológico con tres fases ^(^ 15 ^-^ 17 ^)^ . En la primera, se encuentran las actividades relativas al desarrollo del sistema computacional; en la segunda fase, se realizó la validación de la funcionalidad de identificación de referencias duplicadas; y en la tercera, el test de usabilidad o test en uso fue conducido para identificar la utilidad de la herramienta, el grado de dificultad de uso y los ajustes necesarios para la mejora de la interfaz de usuario. El test en uso se realizó con voluntarios, de manera remota y sincrónica, utilizando la técnica de resolución de tareas, seguida de la aplicación del cuestionario *System Usability Scale* (SUS) ^(^ 18 ^-^ 19 ^)^ . 

### Aspectos éticos

 El proyecto siguió los términos de la Resolución del *Conselho Nacional de Saúde* (CNS 466/12) del Ministerio de Salud de Brasil y fue aprobado por el Comité de Ética de la *Faculdade de Saúde Pública* (FSP) de la *Universidade de São Paulo* (USP), bajo Certificado de Presentación de Apreciación Ética 36397420.8.0000.5421. El Consentimiento Informado (CI) se obtuvo electrónicamente. 

### Sistema “Apoyo a la Revisión Sistemática”

 El sistema *web* “Apoyo a la Revisión Sistemática” (AReS), disponible en la dirección electrónica http://revisaosistematica.com.br , fue propuesto por la autora principal durante el Curso de Doctorado del Programa de Epidemiología de la FSP/USP, en asociación con un profesional de tecnología de la información, que lo construyó voluntariamente. Sus requisitos funcionales se basaron en manuales sobre revisión sistemática, sin embargo, las funcionalidades son genéricas, atendiendo a otros tipos de revisiones, como alcance, *overview* e integrativa. 

 En la versión para computadora ( *desktop* o *notebook* ), el AReS consiste en una página de autenticación que redirige al usuario a la página principal, con un diseño estándar para sitios de administración. De esta manera, el sistema permite la importación de archivos de bases de datos predefinidas como *PubMed* , *Embase* y *Web of Science* , comparando las referencias para identificación de duplicados. Más de un investigador puede participar de la revisión, como revisor principal o invitado. El AReS está adaptado al trabajo en equipo, común en revisiones de literatura, permitiendo la organización y compartimiento de los resúmenes. En la fase de selección de estudios, el sistema permite la lectura de títulos y resúmenes y, después de la ruptura del cegamiento, compara las respuestas de los investigadores sobre la elegibilidad de los estudios, indicando las divergencias ^(^ 6 ^)^ . 

 El modelo incremental utilizado en la construcción del sistema permite que nuevas funcionalidades sean incorporadas en nuevas versiones ^(^ 20 ^)^ . La evaluación de usabilidad incluyó todas las funcionalidades de los menús “Resúmenes”, “Revisiones”, “Grupos”, “Criterios” y “Revisores”. 

### Participantes y lugar del estudio

 La evaluación de usabilidad ocurrió entre marzo y julio de 2022, con la participación de estudiantes de los programas de posgrado *stricto sensu* de la FSP/USP (de los cursos de Maestría Profesional en Entomología en Salud Pública; Doctorado en Epidemiología y en Salud Pública), que formalmente estaban cursando sus estudios en 2022. Las invitaciones fueron enviadas por medio de mensajes electrónicos por la Comisión de Posgrado de la FSP/USP. La muestra por voluntarios incluyó posgraduandos por ser un público con gran interés en la conducción de revisiones y potenciales usuarios del AReS. El estudio se limitó a la FSP para viabilizarlo. 

### Criterios de elegibilidad

 Consistieron en criterios de inclusión: poseer vínculo formal con un programa de posgrado *stricto sensu* de la FSP/USP; tener acceso a un ordenador con internet y navegador actualizado; y concluir al menos el 50% de la etapa de eliminación de estudios duplicados utilizando el sistema computacional. 

### Definición de la muestra

 El tamaño de la muestra siguió lo descrito en la literatura, que orienta una muestra mínima de ocho usuarios ejecutando tareas en un contexto de uso real, para obtener resultados fiables ^(^ 16 ^,^ 21 ^)^ . 

### Tareas y criterios de evaluación

 La evaluación en uso del AReS incluyó dos actividades, organizadas en cuatro etapas y 21 tareas. En la actividad 1, etapas 1, 2 y 3, cada participante asumió el papel de revisor principal, responsable por la creación de una revisión y organización de los resúmenes relacionados al tema antropometría en fibrosis quística. De manera detallada, las tareas eran referentes a la exclusión de referencias duplicadas (etapa 1), selección ciega de estudios (etapa 2), ruptura del cegamiento, comparación de las decisiones en cuanto a la elegibilidad de resúmenes y resolución de divergencias (etapa 3). En la actividad 2, etapa 4, el participante actuó como revisor invitado en una revisión organizada por la investigadora, con el cumplimiento de tareas relativas a la identificación de estudios elegibles ( Figura 1 ). 

**Figura 1:** - Objetivo pretendido en cada etapa y tareas a realizarse en el sistema “Apoyo a la Revisión Sistemática”

*Tareas no obligatorias para la conclusión de la etapa de manera completa y correcta; ^†^ PDF = *Portable Document Format*

 Se registró, para cada participante, la completitud de la tarea, el alcance del resultado esperado y el uso de soporte (consulta a materiales explicativos sobre la utilización del AReS, siendo disponibles vídeos y tutoriales), ocurrencia de errores en el uso debido a problemas de las interfaces del sistema computacional, y tiempo (minutos) para completar cada tarea. El error en el uso fue definido como la acción (o falta de acción) del usuario que llevaba a un resultado diferente del pretendido por el fabricante o por el usuario ^(^ 16 ^-^ 17 ^)^ . 

El tiempo utilizado por el participante para resolver las tareas 9, 10, 11, 19, 20 y 21, que fundamentaban la decisión sobre la elegibilidad de los resúmenes, fue sumado al tiempo utilizado en la resolución de las tareas 8 y 18, referentes a la lectura y análisis de títulos y resúmenes. El cumplimiento de las tareas 5, 8, 13 y 18 fue considerado obligatorio para la definición de conclusión de la etapa, pues, si el participante no las completaba, no sería posible alcanzar los objetivos de exclusión de las referencias duplicadas (etapa 1), selección de estudios (etapas 2 y 4) y resolución de divergencias entre revisores (etapa 3).

### Recolección de datos

 Los datos fueron recolectados individualmente, mediante herramienta de teleconferencia ( *Google Meet* ), iniciándose con el suministro de información y de un enlace para la firma electrónica del CI. La moderadora proporcionó un enlace para la descarga de un archivo en PDF ( *Portable Document Format* ) con instrucciones para acceso al AReS y actividades a ser realizadas. No hubo entrenamiento previo para el uso del sistema computacional. 

 Se solicitó el compartimiento de la pantalla del dispositivo del participante para grabación y posterior verificación de la conclusión de las tareas, necesidad de soporte para resolverlas y ocurrencia de errores en el uso del sistema computacional. Las informaciones fueron registradas por la investigadora en una ficha. El tiempo esperado para la terminación de todas las tareas era de 60 minutos. El tiempo de las acciones y clics efectuados en el AReS fueron registrados en archivo *log* , guardados en banco de datos interno. 

Durante la sesión, fueron aclaradas dudas relacionadas a los enunciados de las tareas, pero no sobre cómo resolverlas. Las instrucciones de uso contenidas en el AReS podían ser consultadas en cualquier momento. Cuando el participante verbalizó que tenía dificultad para realizar una tarea, hubo incentivo para que continuara intentando resolverla, ofreciéndo soporte solo en ausencia de resolución del problema. El soporte consistió en la presentación del enlace para un sitio que contenía materiales explicativos sobre el uso del sistema computacional.

 Tras la conclusión de las tareas, los participantes respondieron dos cuestionarios electrónicos: uno sobre caracterización de los participantes y otro conteniendo la versión en lengua portuguesa del instrumento SUS ^(^ 19 ^)^ para evaluación de usabilidad del AReS. Se solicitó el registro de comentarios sobre su uso y sugerencias de mejoras. 

### Análisis de los datos

 Para la evaluación en uso, se verificó la conclusión de la tarea con o sin suporte (eficacia), el tiempo de resolución (eficiencia) y el grado de usabilidad (instrumento SUS), conforme lo recomendado en normas técnicas ^(^ 16 ^-^ 17 ^)^ . 

 Para la eficacia, se optó por las métricas: ( *i* ) tasa de conclusión de la tarea (porcentaje de participantes que alcanzaron el objetivo de cada tarea de forma completa y correcta), ( *ii* ) tasa de conclusión con soporte (porcentaje de participantes que completaron cada tarea correctamente, con consulta a los materiales explicativos), ( *iii* ) tasa de error en el uso del sistema (porcentaje de tareas con resultados diferentes de lo pretendido debido a problemas en la interfaz) ^(^ 16 ^-^ 17 ^)^ . 

Las acciones no esperadas, identificadas mediante observación no intrusiva, fueron anotadas y permitieron la identificación de los errores en uso y la indicación de los ajustes necesarios en el sistema computacional. Se describieron las frecuencias absoluta y relativa de los resultados relacionados a su eficacia, a los errores en el uso y tipo de material explicativo consultado.

 En relación a la eficiencia, propiedad de calidad que indica los recursos utilizados para la conclusión de las tareas, se optó por medir el tiempo gastado en su resolución como un *proxy* del grado de dificultad encontrado y la consiguiente cantidad de recursos utilizados, como orienta la norma técnica ^(^ 16 ^-^ 17 ^)^ . La medición del tiempo en uso es un *proxy* de la medición de los recursos involucrados porque sería necesario medir el esfuerzo humano para que el sistema computacional produjera los resultados esperados. Tal esfuerzo podría ser indicado por el número de clics, uso de funcionalidades y menús, lo que sería una tarea compleja en esta investigación por involucrar la cuantificación de todas las acciones registradas por el sistema computacional en archivo de texto. 

Los resultados de eficiencia se presentan en tabla, mediante media, desviación estándar, valores mínimo y máximo, así como Intervalo de Confianza del 95% (IC95%) para la media, según etapa.

 El constructo usabilidad, medido mediante el instrumento SUS ^(^ 19 ^)^ , permitió la obtención del puntaje de usabilidad. Este instrumento contiene 10 ítems graduados por medio de escala del tipo Likert, con valores de 1 a 5 correspondientes, respectivamente, a “discrepo totalmente”, “discrepo parcialmente”, “no estoy de acuerdo, ni en desacuerdo”, “de acuerdo parcialmente” y “de acuerdo totalmente”. En la puntuación final, para las preguntas impares, el puntaje se calculó como la nota recibida menos 1, mientras que para las preguntas pares, como 5 menos la nota recibida. Los puntajes pares e impares se sumaron y multiplicaron por 2,5 para obtener la nota final. Las puntuaciones finales se presentaron en categorías de usabilidad: peor imaginable (nota de 0 a 24,9); pobre (de 25,0 a 38,9); mediana (de 39,0 a 51,9); buena (de 52,0 a 73,9); excelente (de 74,0 a 84,9); y mejor imaginable (de 85,0 a 100) ^(^ 22 ^)^ . 

## Resultados

### Caracterización de los participantes

 Participaron 21 estudiantes con una edad media de 39 años (desviación estándar = 7 años; valor mínimo = 28 y máximo = 59 años). Doce (57%) eran del sexo femenino, 20 (95%) eran doctorandos y uno (5%) maestrando. Dieciocho (86%) habían realizado anteriormente algún tipo de estudio de revisión, siendo sistemática (n=9; 53%), narrativa (n=5; 29%) y ambos tipos (n=3; 18%). Seis (33%) reportaron haber utilizado algún tipo de *software* para realizar una revisión, siendo *EndNote* ^®^ el más frecuentemente utilizado, seguido por *Exce* l^®^, *Rayyan* ^®^, *Mendeley* ^®^ y *Word* ^®^. Se excluyeron los individuos que firmaron el CI electrónico, pero no iniciaron las actividades en el sistema computacional o no respondieron a los instrumentos de investigación (n=2). 

### Eficacia

 En el proceso evaluativo, se obtuvo una tasa de conclusión del 90% (n=19 tareas) en las etapas 1 y 3 (referentes a la exclusión de duplicados y resolución de divergencias) y del 95% (n=20) en las etapas 2 y 4 (selección de estudios). En las tareas 14 y 21, las tasas de conclusión fueron inferiores al 90%, sin embargo, no hubo interferencia en el alcance del objetivo de cada etapa. Entre las tareas concluidas, las que presentaron mayor porcentaje de soporte fueron las de número 12 (comparación de las decisiones sobre la elegibilidad de los estudios) y 17 (selección de la revisión en la que fue invitado). El porcentaje de soporte entre los que concluyeron las tareas fue del 9%. La verificación de las tareas que presentaron las menores tasas de conclusión permitió identificar parte de los ajustes a ser realizados en el sistema computacional ( Tabla 1 ). 

 Para las tareas completadas, se produjeron 34 momentos de soporte, siendo acceso a videos explicativos en 28 momentos: tareas 6 (n=1); 8 (n=3); 12 (n=12); 16 (n=3); 17 (n=8) y 18 (n=1), y lectura de tutoriales en seis momentos: tareas 4 (n=1); 12 (n=1); 13 (n=1); 14 (n=1) y 17 (n=2). La elección de los materiales era realizada por el participante basándose en los recursos tecnológicos disponibles, como la calidad de internet, en el momento del test. Los videos también fueron utilizados para soporte en tres tareas no completadas (4, 8, 12). Estos datos no se presentaron en la Tabla 1 , que se refiere solo a las tareas completadas. 

**Tabla 1:** - Distribución de participantes que resolvieron las tareas utilizando el sistema “Apoyo a la Revisión Sistemática”, según las medidas de conclusión de la tarea, soporte y error en el uso. São Paulo, SP, Brasil, 2022

Etapa*	**Tarea**		**Tasa de conclusión**		**Conclusión con** **soporte**			**Error en el uso**	
			n	%	Total	n	%	n	%
1	1.	Crear una revisión	21	100	21	0	-	6	29
	2.	Crear tres grupos para guardar los resúmenes de las bases de datos	21	100	21	0	-	0	-
	3.	Importar los archivos con registros bibliográficos	21	100	21	0	-	0	-
	4.	Identificar las referencias duplicadas	19	90	19	1	5	0	-
	5.	Excluir las duplicatas (4 resúmenes)	19	90	19	0	-	0	-
2	6.	Digitar los criterios de elegibilidad	21	100	21	1	5	0	-
	7.	Incluir un(a) revisor(a)	21	100	21	0	-	0	-
	8.	Leer y analizar título y resumen (4 resúmenes)	20	95	20	3	15	16	80
	9.	Decidir sobre la pertinencia del estudio basado en criterios de elegibilidad	21	100	21	0	-	0	-
	10	Decidir sobre la elegibilidad del estudio	21	100	21	0	-	0	-
	11.	Incluir comentarios, si necesario ^†^	21	100	21	0	-	0	-
3	12.	Comparar las decisiones sobre la elegibilidad de cada estudio	19	90	19	13	68	5	26
	13	Resolver las divergencias entre revisores(as)	19	90	19	1	5	0	-
	14	Utilizar un filtro para identificar los resúmenes elegibles ^†^	12	57	12	1	8	0	-
	15	Abrir un archivo en PDF ^‡^ con resúmenes elegibles ^†^	19	90	19	0	-	0	-
4	16.	Leer la notificación de la revisión en la que fue invitado	21	100	21	3	15	11	52
	17.	Seleccionar la revisión en la que fue invitado	20	95	20	10	55	0	-
	18.	Leer y analizar título y resumen	20	95	20	1	5	8	40
	19.	Decidir sobre la pertinencia del estudio basado en criterios de elegibilidad	20	95	20	0	-	0	-
	20.	Decidir sobre la elegibilidad del estudio	20	95	20	0	-	0	-
	21.	Incluir comentarios, si necesario ^†^	15	71	15	0	-	0	-
**Total**			**411**	**93**	**411**	**34**	**9**	**46**	**11**

* Etapa: 1) Eliminar duplicados; 2) Identificar estudios elegibles; 3) Resolver divergencias entre revisores; 4) Identificar estudios elegibles como revisor invitado

^†^ Tareas no obligatorias para la conclusión de la etapa de manera completa y correcta

^‡^ PDF = *Portable Document Format*

 Durante la resolución, surgieron errores en el uso para las tareas que fueron completadas ( Tabla 1 ) y no completadas, como la 4 (n=4), 8 (n=1), 12 (n=1) y 13 (n=1). Estas ocurrencias permitieron identificar los ajustes necesarios en el AReS, dependiendo del tipo de error y de la consecuencia de su ocurrencia, pudiendo (o no) interferir en su eficacia o eficiencia. El detalle de los errores en uso observados y el resultado del análisis se presentan en la Figura 2 . 

**Figura 2:** - Acciones realizadas por los participantes que interfirieron en las propiedades de calidad del sistema “Apoyo a la Revisión Sistemática” o problemas en la interfaz del sistema computacional que no permitieron el resultado deseado

### Eficiencia

 La utilización del AReS requirió un tiempo total medio de 55,1 minutos (desviación estándar=15,8). Para la resolución de las tareas, según el objetivo pretendido en cada etapa, el tiempo medio varió de 12,4 a 15,8 minutos ( Tabla 2 ). 

**Tabla 2:** - Tiempo medio (minutos) para la conclusión de las etapas utilizando el sistema “Apoyo a la Revisión Sistemática” (n = 21). São Paulo, SP, Brasil, 2022

**Etapa/objetivo**		**Tiempo medio** **(minutos)**	DE*	**Mediana**	**Mínimo** **m** áximo	**IC** ^†^ **95%**
1	Exclusión de los registros duplicados	12,8	7,0	11,7	4-31	10-16
2	Identificación de estudios elegibles	15,8	6,8	14,9	7-32	13-19
3	Resolución de divergencias	14,0	8,1	12,8	1-32	10-18
4	Selección de estudios elegibles, como invitado	12,4	4,2	12,9	4-20	11-14
Total del proceso evaluativo		55,1	15,8	55,1		

* DE = Desviación-estándar

^†^ IC95% = Intervalo de confianza de 95%

### Usabilidad del AReS

 La nota final media de usabilidad del AReS resultó en 82,4; mediana 85; valores mínimo y máximo 35 y 97,5 (en escala de 0 a 100). La clasificación del resultado de este constructo por participante fue: “mejor imaginable” (n=12; 57%), “excelente” (n=5; 24%), “bueno” (n=3; 14%) y “pobre” (n=1; 5%). En la Figura 3 , se presentan las frecuencias, las notas finales medias y el IC95%, según las categorías de usabilidad en el uso del sistema computacional. Se observa que no se presentó la media y el IC95% para la categoría “pobre”, ya que tiene un participante. A través del IC95%, se puede afirmar que la categoría “mejor imaginable” se destaca cuando se compara con las categorías “bueno” y “excelente”, siendo esta última la categoría de mayor representación de participantes ( Figura 3 ). 

*IC95% = Intervalo de confianza de 95%

**Figura 3:** - Distribución de participantes, según puntuación (A) y nota media por categoría de usabilidad (B) del instrumento *System Usability Scale* para el sistema “Apoyo a la Revisión Sistemática”

#### Condiciones generales observadas durante la prueba de uso del AReS

 El sistema operativo más utilizado fue *Microsoft Windows* con el navegador *Chrome* (n=18), seguido de *MacOS* con navegador *Safari* (n=2). Se observaron errores en *Safari* , como la no importación de archivos y la selección automática de decisión sobre la elegibilidad de los estudios, mostrando la necesidad de ajustes en el AReS. Durante la prueba, los participantes comenzaron a utilizar el navegador *Chrome* para un mejor uso del sistema computacional y para completar las tareas 3 y 8. 

La cámara fue mantenida abierta por la mayoría de los participantes (n=14). Uno de ellos dijo que la mantendría cerrada debido a problemas en su funcionamiento. Se solicitó al participante que “pensara en voz alta”, y nueve (43%) siguieron esa instrucción. La mayoría de los participantes compartieron la pantalla entera, sin embargo, cuando solo se compartía la pestaña de uso del AReS, la investigadora no podía verificar el tipo de material explicativo utilizado.

 Se necesitó reprogramar la prueba para dos participantes por motivos de lentitud en internet y problemas en su computadora. El sistema AReS presentó problemas al presionar el ícono para seleccionar la revisión en la que el participante fue invitado (Tarea 17), siendo necesario presionar el ícono actualizar o hacer nuevo *login* . En la actividad 2 (revisor invitado), el sistema computacional mostró lentitud y problemas técnicos que impidieron que algunos participantes concluyeran la Tarea 21. 

Algunos participantes hicieron sugerencias: usar resúmenes en lengua portuguesa en la próxima prueba (n=3) y dejar claro, al inicio, que la participación sería como revisor principal y luego como invitado (n=1). Un participante verbalizó una queja sobre el gran número de resúmenes que deberían ser leídos (n=8 resúmenes) y otro verbalizó que el PDF con las tareas no explicaba cómo resolverlas, incluso después de la exposición de la investigadora de que el participante debería leer la tarea e intentar resolverla solo en el sistema computacional.

#### Comentarios de los participantes

Las sugerencias de los participantes sobre el AReS se presentan a continuación:

Mejorar el cuadro de importación de las bases de datos (...) para inducir la selección de archivo de importación (P1).

Incluir un ícono de orientación sobre la atención a los detalles de la pantalla y explicación de cada ítem (P2).

Encontré que el sistema es bastante intuitivo. Sin embargo, no identifiqué cómo hacer el cambio de elegibilidad cuando hay divergencia entre los autores. El gráfico que está en el dashboard solo me mostró el porcentaje de trabajos elegibles para cada autor, dando una falsa impresión de no haber divergencias. Pero, al mirar de nuevo, había divergencias (P3).

Creo que la interfaz con el usuario es difícil y poco intuitiva, es difícil descubrir el próximo paso. Creo que faltan cuadros informativos sobre cómo operar el sistema o cómo realizar determinada tarea. No sé cómo sería si hubiera tenido un entrenamiento previo sobre el sistema, cómo sería la capacidad de uso, pero, como las pruebas fueron sin un entrenamiento completo (solo algunos datos de manual), sentí bastante dificultad (P4).

### Ajustes y mejoras

 Entre los ajustes y mejoras, se incluyó: estandarizar, en todos los navegadores, el texto del cuadro de selección de archivos para *upload* ; incluir globos explicativos sobre los botones de las pantallas del sistema computacional; incluir botón para romper el cegamiento, para facilitar la visualización de la comparación de las respuestas de los revisores; permitir la selección de una revisión por el menú “Revisiones” (y no solo por la barra superior); listar, separadamente, las revisiones creadas por el propio investigador y las revisiones en las que fue invitado a participar; en la pantalla de resúmenes, exhibir el botón guardar solo cuando haya inclusión de un comentario; permitir hacer clic en el título del estudio para acceder al resumen; en la identificación de duplicados, eliminar la opción de seleccionar grupo para comparación de las referencias. 

## Discusión

 El presente estudio evaluó el sistema “Apoyo a la Revisión Sistemática” (AReS), que presenta funcionalidades para las fases iniciales de estudios de revisión de literatura, estructurado de acuerdo con una metodología rigurosa ^(^ 1 ^,^ 3 ^)^ , siguiendo también los requisitos de normativa internacional ^(^ 16 ^)^ . La evaluación empírica mostró un alto grado de usabilidad percibida por parte de los evaluadores voluntarios, quienes eran estudiantes de posgrado y que, no intencionalmente, constituyeron un grupo heterogéneo en cuanto al uso de herramientas computacionales en estudios de revisión, siendo representativos del público objetivo. 

 Siempre se debe considerar la posibilidad de sesgo de selección al utilizar muestra por voluntarios, ya que la motivación por el *software* pudo haber sido por el interés en conocer la nueva herramienta o por la oportunidad de aprender sobre su uso ^(^ 22 ^)^ . La segunda opción es corroborada por el porcentaje del 67% de participantes que reportaron no haber utilizado anteriormente ese tipo de herramienta. Tal característica pudo haber provocado un efecto positivo en el estudio, ya que podrían necesitar más recursos computacionales y exigido más del sistema en evaluación ^(^ 23 ^)^ . 

La opción metodológica por no entrenamiento previo permitió la exposición a un nuevo producto y favoreció el surgimiento de información relativa a las dificultades de uso debido a las interfaces del sistema computacional, otorgando al test autenticidad y cumplimiento del objetivo del estudio.

 Las tareas propuestas en el test en uso fueron creadas pensando en un paso a paso ^(^ 24 ^)^ para la realización de una revisión, siguiendo una secuencia en cuatro etapas, que favorecía la verificación de la eficacia, eficiencia y el monitoreo de los problemas de interfaz. Como la calidad de una revisión depende de una selección no sesgada de estudios ^(^ 3 ^)^ , la estrategia de más de un evaluador fue contemplada en el AReS, resultando en una evaluación más larga, hecha en dos fases, cada una con roles diferentes para el revisor (principal e invitado). 

Los participantes alcanzaron los objetivos de las etapas en altos porcentajes, con una tasa de conclusión de al menos el 90%, siendo consideradas satisfactorias por los autores, otorgando al proceso evaluativo la calidad de validez interna, indicando que los recursos metodológicos utilizados fueron capaces de captar la adecuación de las funcionalidades del AReS.

 Para las tareas 14 (uso de filtro para identificar los resúmenes elegibles) y 21 (inclusión de comentarios, si el estudio es considerado no elegible por el revisor invitado) que tuvieron tasas de conclusión inferiores al 90%, la influencia en el desempeño del *software* es mínima, indicando por medio de la tarea 14 un posible problema en la especificación de la tarea, y no en la interfaz. Con la segunda ocurrencia, se puede identificar necesidad de corrección de problemas técnicos en el sistema. Solo la tarea 12 fue considerada compleja, pues involucraba acceso a más de un menú para alcanzar la conclusión, justificando el soporte requerido por la mayoría de los participantes e indicación de necesidad de inclusión de un botón de acceso directo. 

 El tiempo medio del test de usabilidad varió conforme lo esperado por los investigadores. En situación de conducción de una investigación, el tiempo medio de lectura y análisis de títulos y resúmenes (tarea 8) varía conforme la cantidad de referencias recuperadas en las bases de datos, mientras que el tiempo para resolución de las divergencias puede variar según la cantidad de resúmenes a ser comparados ^(^ 25 ^)^ . Los hallazgos del presente estudio corroboran con la literatura que indica que las fases iniciales de revisiones son las que requieren mayor tiempo de trabajo para la conclusión de la selección de estudios elegibles, así, la preferencia es por recursos que favorezcan reducción del tiempo de dedicación a esa etapa ^(^ 13 ^)^ , en relación al trabajo manual y herramientas no específicas, como hojas de cálculo. 

 El AReS fue evaluado con un grado de usabilidad positivo, con el “mejor imaginable” en el 57% de las evaluaciones; 24% como “excelente” y 14% como “bueno”. El cuestionario SUS fue de fácil aplicación, siendo un instrumento validado y ampliamente utilizado en investigaciones y en pruebas conducidas por profesionales de tecnología de la información, incluyendo el área de la salud ^(^ 26 ^)^ . Se destaca que el instrumento SUS también permite la comparación de diferentes sistemas *web* ^(^ 27 ^)^ , con usuarios en diferentes regiones geográficas ^(^ 28 ^)^ . 

 La ocurrencia de errores en el uso, como la no identificación de todas las duplicidades, en general, indicó la necesidad de mayor comunicación entre el sistema computacional y el usuario, resuelta mediante ajustes en la interfaz e inclusión de mensajes explicativos. Identificar los problemas en uso y corregirlos confiere al producto un uso más intuitivo y una mayor adhesión del público objetivo ^(^ 29 ^)^ . 

La prueba realizada de manera remota ya está descrita en la literatura y fue importante porque permitió al participante utilizar sus recursos tecnológicos, posibilitando la prueba de usabilidad en contexto real de uso. Además, permitió la grabación de la sesión de trabajo y el posterior registro de datos, como la identificación de la completitud, el cumplimiento del objetivo de la tarea y la ocurrencia de errores en el uso. El uso de herramientas para teleconferencia fue fundamental y permitió la interacción entre los involucrados, facilitó el compartimiento de materiales instructivos de las actividades, la observación y grabación de las pantallas, sin la necesidad de costos adicionales con impresión de materiales y equipos para filmación.

 Aunque existen diferencias en la conectividad intramunicipal ^(^ 30 ^)^ , las condiciones para la conducción de la prueba fueron consideradas adecuadas por los investigadores. Según la literatura, la prueba remota síncrona no difiere significativamente de las pruebas realizadas en laboratorio ^(^ 31 ^)^ y favorece la participación de personas ubicadas en diferentes regiones del país o del extranjero, pudiendo ser preferida en investigaciones con bajo presupuesto para este tipo de evaluación y demandas específicas, como el aislamiento social impuesto por la pandemia de COVID-19. 

 Como potencialidad de este estudio, era mandatorio realizar la prueba de usabilidad conducida según procedimientos metodológicos reconocidos, para disponibilidad de un *software* confiable, eficaz y con alto grado de usabilidad. Las tecnologías que producen resultados precisos son cruciales para ayudar en la toma de decisiones y asegurar la seguridad y exactitud de los datos. Incluir detalladamente en este trabajo el proceso evaluativo del producto puede ayudar a los profesionales de la salud que participan en otros procesos evaluativos, pues presenta herramientas específicas para la evaluación de un *software* , fundamenta las elecciones de uso y explica las propiedades de calidad a ser evaluadas. 

 Las características importantes para la elección de programas computacionales específicos en revisión sistemática son la simplicidad, la facilidad de aprendizaje y la intuitividad del diseño ^(^ 12 ^)^ . Se buscó, en la construcción del producto presentado, alcanzar tales atributos. Para controlar el sesgo en la selección de estudios elegibles, se incorporó en el AReS la posibilidad de participación de más de un evaluador y el cegamiento de las decisiones, asegurando la independencia entre sus decisiones. Otra característica del AReS es la presentación, en una sola pantalla, del resumen del artículo y de los criterios de elegibilidad que, al ser completados, conducen a la decisión de ser elegibles (o no). Esta funcionalidad no fue encontrada en ningún otro sistema, siendo una propuesta original y única del AReS. 

Las limitaciones del estudio se refieren al motivo de la construcción del AReS, que tuvo origen en la necesidad específica de la investigadora en la operacionalización de una revisión. Se presenta solo en lengua portuguesa, incluyendo los materiales de apoyo. El instrumento utilizado para la evaluación de usabilidad (SUS) está validado para la lengua portuguesa en Portugal; en la planificación de la prueba, no se recuperó estudio detallado de adaptación transcultural de este instrumento para el contexto brasileño.

Además, como limitación metodológica, se puede mencionar la no homogeneidad del procedimiento de apertura de las cámaras durante la prueba en uso, sin embargo, tal hecho parece no haber interferido en los resultados, ya que no era de interés evaluar la dimensión comportamental en el uso de la herramienta. El no compartimiento de la pantalla durante el uso de material explicativo también fue un factor limitante. No obstante, se destaca que la investigadora solicitaba esa información para registro en ficha. El no esclarecimiento a un participante sobre el papel que iba a desempeñar como revisor principal o invitado puede considerarse como una falla metodológica de la prueba. Esta ocurrencia fue verbalizada por el segundo participante y las orientaciones pasaron a ser dadas a todos los demás.

 En comparación con otras herramientas computacionales, la presente versión del AReS posee funcionalidades para la eliminación de duplicados y selección de estudios, a diferencia de aquellas que apoyan otras fases de una revisión sistematizada, como la extracción de datos, ayudada por *DistillerSR* ^(^ 32 ^)^ . El AReS no resalta palabras clave, ni permite la importación de artículos completos, como el *Rayyan* . 

 Como visión de futuro, se puede destacar que el desarrollo de funcionalidades para otras etapas de revisión, utilizando aplicaciones de inteligencia artificial, así como otras herramientas computacionales disponibles ^(^ 33 ^-^ 34 ^)^ , podría ampliar su uso por estudiantes, investigadores y profesionales del área de la salud. 

## Conclusión

 El AReS presenta parámetros de eficacia y eficiencia necesarios para una herramienta computacional de revisión. Presenta usabilidad de 82,4 en escala de 0 a 100 evaluada por el instrumento *System Usability Scale* (SUS). La versión presentada incorpora los ajustes identificados en el test en uso. El AReS es una herramienta útil y de fácil utilización, orientada al medio académico y de investigación, propuesta para disminuir el tiempo de trabajo en fases iniciales de revisiones de literatura. 

# Translation: Usabilidade do sistema computacional “Apoio à Revisão Sistemática”: um estudo metodológico *

**DOI:** 10.1590/1518-8345.7081.4375

**Published:** 2024-10-07

**Authors:** Fernanda Martins Dias Escaldelai, Leandro Escaldelai, Denise Pimentel Bergamaschi

**Affiliations:** ^1^ Universidade de São Paulo, Faculdade de Saúde Pública, São Paulo, SP, Brasil.; ^2^ Faculdade de Tecnologia de São Paulo, São Paulo, SP, Brasil.

**Keywords:** Computação em Nuvem, Design Centrado no Usuário, Revisão, Revisão Sistemática, Software, Tecnologia da Informação

## Abstract

avaliar a usabilidade do sistema computacional “Apoio à Revisão Sistemática”.

estudo metodológico envolvendo 21 estudantes, que avaliaram o sistema computacional em um teste de usabilidade remoto e síncrono. Foram realizadas duas atividades, contendo 21 tarefas simulando as etapas iniciais de um estudo de revisão, propostas para eliminação de referências duplicadas, seleção de estudos e resolução de divergências entre avaliadores. No final, os participantes responderam ao questionário *System Usability Scale* . As medidas utilizadas foram: taxa de conclusão com assistência (eficácia) e tempo para concluir a tarefa (eficiência). Os dados foram descritos em tabela por meio de média, desvio-padrão, valores mínimo e máximo.

no total de 21 tarefas, os participantes apresentaram taxa de conclusão com assistência de 9% e tempo médio de 55,1 minutos (sendo 60 minutos o tempo esperado). A nota média fornecida pelo instrumento *System Usability Scale* foi 82,4 (considerando uma pontuação de 0 a 100), mediana 85; valor mínimo 35 e máximo 97,5.

o sistema computacional apresenta parâmetros de eficácia e eficiência necessários a uma ferramenta computacional de revisão. Apresenta usabilidade de 82,4 em escala de 0 a 100 avaliada pelo instrumento *System Usability Scale* . A versão apresentada incorpora os ajustes identificados no teste em uso.

## Destaques

**(1)** O sistema “Apoio à Revisão Sistemática” possui alto grau de usabilidade percebida.

**(2)** Seu uso facilita a eliminação de referências duplicadas e a seleção de estudos.

**(3)** O teste de usabilidade remoto e síncrono viabilizou a pesquisa durante a pandemia.

**(4)** Associar técnicas de usabilidade permitiu identificar os ajustes necessários.

**(5)** O método utilizado ampliou a capacidade do teste em contexto real de uso.

## Introdução

 Os estudos de revisão que fornecem uma síntese imparcial das evidências científicas incluem a seleção de estudos baseada em métodos rigorosos para permitir resultados válidos e reprodutíveis ^(^ 1 ^)^ . A etapa inicial, a qual inclui levantamentos em bases de dados, eliminação de artigos duplicados e identificação de estudos elegíveis, é árdua, sobretudo em razão do volume de estudos a serem manuseados, sendo fortemente recomendada a utilização de ferramentas computacionais, como as disponibilizadas na plataforma *The Systematic Review Toolbox* ^(^ 2 ^)^ . 

 Apesar da *Cochrane* recomendar ferramentas computacionais para revisões, como *Covidence, EPPI-Reviewer* e *EndNote* ^®(3)^ , existem outros produtos com essa finalidade ^(^ 2 ^)^ , como o *Rayyan* ^®^ , o *Parsifal* , o *StArt* e o sistema “Apoio à Revisão Sistemática” (AReS), desenvolvido recentemente para auxiliar nas etapas iniciais de revisões na área da saúde. Pode-se encontrar, como limitadores de ferramentas computacionais, o acesso nem sempre gratuito, a interface não amigável, a necessidade de conhecimento em programação e a falta de suporte técnico ^(^ 4 ^)^ . O AReS supera tais dificuldades e apresenta outras vantagens, como interface em língua portuguesa, identificação acurada de duplicatas sem necessidade do trabalho manual ^(^ 5 ^)^ e apoio à seleção de estudos elegíveis, apresentando em uma só tela o resumo e os critérios de elegibilidade cadastrados pelo pesquisador ^(^ 6 ^)^ . 

 Recentemente, houve a necessidade de elaboração de sínteses de evidências científicas para o enfrentamento rápido da pandemia de *coronavirus* 2019 (COVID-19) no desenvolvimento de novas tecnologias para diagnóstico, tratamento e controle da doença ^(^ 7 ^)^ . A urgência por informação atualizada induziu à inovação e ao uso de automação para reduzir o tempo de conclusão das etapas de revisão ^(^ 8 ^)^ e para a avaliação das novas ferramentas, com uso do método de usabilidade remoto para continuidade das pesquisas e obtenção de avanços em tecnologia ^(^ 9 ^)^ . 

 Existe consenso tanto na literatura científica como em normas de boa qualidade técnica quanto à necessidade da avaliação de usabilidade de novos produtos tecnológicos. A usabilidade se refere ao grau com que um produto ou serviço pode ser utilizado por usuários específicos para atingir os objetivos especificados com eficácia, eficiência e satisfação em um determinado contexto de uso ^(^ 2020 ^)^ . 

 A percepção de usabilidade qualificada como baixa é um dos motivos para desistência de utilização de ferramentas para automação de revisões de literatura ^(^ 11 ^)^ . Estudos de usabilidade com realização de tarefas pelos usuários destacaram que a adesão ao uso de *softwares* de revisão depende da facilidade de utilização, qualidade da interface de usuário, recursos e funcionalidades disponíveis ^(^ 12 ^-^ 14 ^)^ . 

Considerando o exposto, o objetivo deste estudo foi avaliar a usabilidade do sistema computacional “Apoio à Revisão Sistemática”.

## Método

### Tipo e delineamento do estudo

 Estudo metodológico com três fases ^(^ 15 ^-^ 17 ^)^ . Na primeira, encontram-se as atividades relativas ao desenvolvimento do sistema computacional; na segunda fase, foi realizada a validação da funcionalidade de identificação de referências duplicadas; e na terceira, o teste de usabilidade ou teste em uso foi conduzido para identificar a utilidade da ferramenta, o grau de dificuldade de uso e os ajustes necessários para a melhoria da interface de usuário. O teste em uso foi realizado com voluntários, de maneira remota e síncrona, utilizando-se a técnica de resolução de tarefas, seguida da aplicação do questionário *System Usability Scale* (SUS) ^(^ 18 ^-^ 19 ^)^ . 

### Aspectos éticos

O projeto seguiu os termos da Resolução do Conselho Nacional de Saúde (CNS 466/12) do Ministério da Saúde do Brasil e foi aprovado pelo Comitê de Ética da Faculdade de Saúde Pública (FSP) da Universidade de São Paulo (USP), sob Certificado de Apresentação de Apreciação Ética 36397420.8.0000.5421. O Termo de Consentimento Livre e Esclarecido (TCLE) foi obtido eletronicamente.

### Sistema “Apoio à Revisão Sistemática”

 O sistema *web* “Apoio à Revisão Sistemática” (AReS), disponível pelo endereço eletrônico http://[revisaosistematica.com.br ]( http://revisaosistematica.com.br ), foi proposto pela autora principal durante Curso de Doutorado do Programa de Epidemiologia da FSP/USP, em parceria com profissional de tecnologia de informação, que o construiu voluntariamente. Seus requisitos funcionais foram baseados em manuais sobre revisão sistemática, entretanto, as funcionalidades são genéricas, atendendo a outros tipos de revisões, como escopo, *overview* e integrativa. 

 Na versão para computador ( *desktop* ou *notebook* ), o AReS consiste em uma página de autenticação que redireciona o usuário para a página principal, com *layout* padronizado para *sites* de administração. Desse modo, o sistema permite a importação de arquivos de bases de dados pré-definidas como *PubMed* , *Embase* e *Web of Science* , comparando as referências para identificação de duplicatas. Mais de um pesquisador pode participar da revisão, como revisor principal ou convidado. O AReS é adaptado ao trabalho em equipe, comum em revisões de literatura, permitindo a organização e compartilhamento dos resumos. Na fase de seleção de estudos, o sistema permite a leitura de títulos e resumos e, após quebra de cegamento, compara as respostas dos pesquisadores sobre a elegibilidade dos estudos, indicando as divergências ^(^ 6 ^)^ . 

 O modelo incremental utilizado na construção do sistema permite que novas funcionalidades sejam incorporadas em novas versões ^(^ 20 ^)^ . A avaliação de usabilidade incluiu todas as funcionalidades dos menus “Resumos”, “Revisões”, “Grupos”, “Critérios” e “Revisores”. 

### Participantes e local de estudo

 A avaliação de usabilidade ocorreu entre março e julho de 2022, com a participação de estudantes dos programas de pós-graduação *stricto sensu* da FSP/USP (dos cursos de Mestrado Profissional em Entomologia em Saúde Pública; Doutorado em Epidemiologia e em Saúde Pública), que formalmente estavam frequentando seus cursos em 2022. Os convites foram enviados por meio de mensagens eletrônicas pela Comissão de Pós-Graduação da FSP/USP. A amostra por voluntários incluiu pós-graduandos, por serem um público com grande interesse na condução de revisões e potenciais usuários do AReS. O estudo ficou restrito à FSP para viabilizá-lo. 

### Critérios de elegibilidade

 Consistiram em critérios de inclusão: possuir vínculo formal com um programa de pós-graduação *stricto sensu* da FSP/USP; ter acesso a um computador com internet e navegador atualizado; e concluir pelo menos 50% da etapa de eliminação de estudos duplicados utilizando o sistema computacional. 

### Definição da amostra

 O tamanho da amostra seguiu o descrito na literatura, que orienta uma amostra mínima de oito usuários executando tarefas em um contexto de uso real, para obtenção de resultados confiáveis ^(^ 16 ^,^ 21 ^)^ . 

### Tarefas e critérios de avaliação

 A avaliação em uso do AReS incluiu duas atividades, organizadas em quatro etapas e 21 tarefas. Na atividade 1, etapas 1, 2 e 3, cada participante assumiu o papel de revisor principal, responsável pela criação de uma revisão e organização dos resumos relacionados ao tema antropometria em fibrose cística. De modo detalhado, as tarefas eram referentes à exclusão de referências duplicadas (etapa 1), seleção cega de estudos (etapa 2), quebra do cegamento, comparação das decisões quanto à elegibilidade de resumos e resolução de divergências (etapa 3). Na atividade 2, etapa 4, o participante atuou como revisor convidado em uma revisão organizada pela pesquisadora, com o cumprimento de tarefas relativas à identificação de estudos elegíveis ( Figura 1 ). 

**Figura 1:** - Objetivo pretendido em cada etapa e tarefas a serem realizadas no sistema “Apoio à Revisão Sistemática”

*Tarefas não obrigatórias para conclusão da etapa de maneira completa e correta; ^†^ PDF = *Portable Document Format*

 Registrou-se, para cada participante, a completude da tarefa, o alcance do resultado esperado e uso de assistência (consulta a materiais explicativos sobre a utilização do AReS, sendo disponibilizados vídeos e tutoriais), ocorrência de erros no uso decorrentes de problemas das interfaces do sistema computacional, e tempo (minutos) para completar cada tarefa. O erro no uso foi definido como a ação (ou falta de ação) do usuário que levava a um resultado diferente do pretendido pelo fabricante ou pelo usuário ^(^ 16 ^-^ 17 ^)^ . 

O tempo utilizado pelo participante para resolver as tarefas 9, 10, 11, 19, 20 e 21, que fundamentavam a decisão sobre a elegibilidade dos resumos, foi somado ao tempo utilizado na resolução das tarefas 8 e 18, referentes à leitura e análise de títulos e resumos. O cumprimento das tarefas 5, 8, 13 e 18 foi considerado obrigatório para a definição de conclusão da etapa, pois, se o participante não as completasse, não seria possível alcançar os objetivos de exclusão das referências duplicadas (etapa 1), seleção de estudos (etapas 2 e 4) e resolução de divergências entre revisores (etapa 3).

### Coleta de dados

 Os dados foram coletados individualmente, por meio de ferramenta de teleconferência ( *Google Meet* ), iniciando-se com o fornecimento de informações e de um *link* para a assinatura eletrônica do TCLE. A moderadora forneceu um *link* para *download* de um arquivo em PDF ( *Portable Document Format* ) com instruções para acesso ao AReS e atividades a serem realizadas. Não houve treinamento prévio para uso do sistema computacional. 

 Foi solicitado o compartilhamento da tela do dispositivo do participante para gravação e posterior verificação da conclusão das tarefas, necessidade de assistência para resolvê-las e ocorrência de erros no uso do sistema computacional. As informações foram registradas pela pesquisadora em uma ficha. O tempo esperado para término de todas as tarefas era de 60 minutos. O tempo das ações e cliques efetuados no AReS foram registrados em arquivo *log,* salvos em banco de dados interno. 

 Durante a sessão, foram esclarecidas dúvidas relacionadas aos enunciados das tarefas, mas não sobre como resolvê-las. As instruções de uso contidas no AReS poderiam ser consultadas a qualquer momento. Quando o participante verbalizou que havia dificuldade para realização de uma tarefa, houve incentivo para que continuasse tentando resolvê-la, sendo oferecida assistência somente na ausência de resolução do problema. A assistência consistiu na apresentação do *link* para um *site* que continha os materiais explicativos sobre o uso do sistema computacional. 

 Após a conclusão das tarefas, os participantes responderam dois questionários eletrônicos: um sobre caracterização dos participantes e outro contendo a versão em língua portuguesa do instrumento SUS ^(^ 19 ^)^ para avaliação de usabilidade do AReS. Solicitou-se o registro de comentários sobre o seu uso e sugestões de melhorias. 

### Análise dos dados

 Para avaliação em uso, verificou-se a conclusão da tarefa com ou sem assistência (eficácia), o tempo de resolução (eficiência) e o grau de usabilidade (instrumento SUS), conforme o recomendado em normas técnicas ^(^ 16 ^-^ 17 ^)^ . 

 Para a eficácia, optou-se pelas métricas: ( *i* ) taxa de conclusão da tarefa (percentual de participantes que atingiram o objetivo de cada tarefa de forma completa e correta), ( *ii* ) taxa de conclusão com assistência (percentual de participantes que completaram cada tarefa corretamente, com consulta aos materiais explicativos), ( *iii* ) taxa de erro no uso do sistema (percentual de tarefas com resultados diferentes do pretendido em razão de problemas na interface) ^(^ 16 ^-^ 17 ^)^ . 

As ações não esperadas, identificadas por meio de observação não intrusiva, foram anotadas e permitiram a identificação dos erros em uso e a indicação dos ajustes necessários no sistema computacional. Foram descritas as frequências absoluta e relativa dos resultados relacionados à sua eficácia, aos erros no uso e tipo de material explicativo consultado.

 Em relação à eficiência, propriedade de qualidade que indica os recursos utilizados para a conclusão das tarefas, optou-se por aferir o tempo gasto na sua resolução como uma *proxy* do grau de dificuldade encontrado e a consequente quantidade de recursos utilizados, como orientado pela norma técnica ^(^ 16 ^-^ 17 ^)^ . A aferição do tempo em uso é uma *proxy* da aferição dos recursos envolvidos porque seria necessário mensurar o esforço humano para que o sistema computacional produzisse os resultados esperados. Tal esforço poderia ser indicado pelo número de cliques, uso de funcionalidades e menus, o que seria uma tarefa complexa nesta pesquisa por envolver a quantificação de todas as ações registradas pelo sistema computacional em arquivo de texto. 

Os resultados de eficiência são apresentados em tabela, por meio de média, desvio-padrão, valores mínimo e máximo, bem como Intervalo de Confiança de 95% (IC95%) para a média, segundo etapa.

 O construto usabilidade, aferido por meio do instrumento SUS ^(^ 19 ^)^ , permitiu a obtenção do escore de usabilidade. Esse instrumento contém 10 itens graduados por meio de escala do tipo Likert, com valores de 1 a 5 correspondentes, respectivamente, a “discordo fortemente”, “discordo parcialmente”, “não concordo, nem discordo”, “concordo parcialmente” e “concordo totalmente”. Na pontuação final, para as perguntas ímpares, o escore foi calculado como a nota recebida menos 1, já para as perguntas pares, como 5 menos a nota recebida. Os escores pares e ímpares foram somados e multiplicados por 2,5 para obtenção da nota final. As pontuações finais foram apresentadas em categorias de usabilidade: pior imaginável (nota de 0 a 24,9); pobre (de 25,0 a 38,9); mediana (de 39,0 a 51,9); boa (de 52,0 a 73,9); excelente (de 74,0 a 84,9); e melhor imaginável (de 85,0 a 100) ^(^ 22 ^)^ . 

## Resultados

### Caracterização dos participantes

 Participaram 21 estudantes com idade média de 39 anos (desvio-padrão=7 anos; valor mínimo=28 e máximo=59 anos). Doze (57%) eram do sexo feminino, 20 (95%) eram doutorandos e um (5%) mestrando. Dezoito (86%) haviam realizado anteriormente algum tipo de estudo de revisão, sendo sistemática (n=9; 53%), narrativa (n=5; 29%) e ambos os tipos (n=3; 18%). Seis (33%) relataram ter utilizado algum tipo de *software* para realizar uma revisão, sendo o *EndNote* ^®^ o mais frequentemente utilizado, seguido por *Excel* ^®^ *,* *Rayyan* ^®^ , *Mendeley* ^®^ e *Word* ^®^ *.* Foram excluídos os indivíduos que assinaram o TCLE eletrônico, mas não iniciaram as atividades no sistema computacional ou não responderam aos instrumentos de pesquisa (n=2). 

### Eficácia

 No processo avaliativo, obteve-se taxa de conclusão de 90% (n=19 tarefas) nas etapas 1 e 3 (referentes à exclusão de duplicatas e resolução de divergências) e 95% (n=20) nas etapas 2 e 4 (seleção de estudos). Nas tarefas 14 e 21, as taxas de conclusão foram inferiores a 90%, entretanto, não houve interferência no alcance do objetivo de cada etapa. Entre as tarefas concluídas, as que apresentaram maior percentual de assistência foram as de número 12 (comparação das decisões sobre a elegibilidade dos estudos) e 17 (seleção da revisão em que foi convidado). O percentual de assistência entre os que concluíram as tarefas foi de 9%. A verificação das tarefas que apresentaram as menores taxas de conclusão permitiu identificar parte dos ajustes a serem realizados no sistema computacional ( Tabela 1 ). 

 Para as tarefas concluídas, ocorreram 34 momentos de assistências, sendo acesso a vídeos explicativos em 28 momentos: tarefas 6 (n=1); 8 (n=3); 12 (n=12); 16 (n=3); 17 (n=8) e 18 (n=1), e leitura de tutoriais em seis momentos: tarefas 4 (n=1); 12 (n=1); 13 (n=1); 14 (n=1) e 17 (n=2). A escolha dos materiais era feita pelo participante com base nos recursos tecnológicos disponíveis, como qualidade de internet, no momento do teste. Os vídeos também foram utilizados para assistência a três tarefas não concluídas (4, 8, 12). Esses dados não foram apresentados na Tabela 1 , que se refere apenas às tarefas concluídas. 

**Tabela 1:** - Distribuição de participantes que resolveram as tarefas utilizando o sistema “Apoio à Revisão Sistemática”, segundo as medidas de conclusão da tarefa, assistência e erro no uso. São Paulo, SP, Brasil, 2022

Etapa*	**Tarefa**		**Taxa de conclusão**		**Conclusão com assistência**			**Erro no uso**	
			n	%	Total	n	%	n	%
1	1.	Criar uma revisão	21	100	21	0	-	6	29
	2.	Criar três grupos para salvar os resumos das bases de dados	21	100	21	0	-	0	-
	3.	Importar os arquivos com registros bibliográficos	21	100	21	0	-	0	-
	4.	Identificar as referências duplicadas	19	90	19	1	5	0	-
	5.	Excluir as duplicatas (4 resumos)	19	90	19	0	-	0	-
2	6.	Digitar os critérios de elegibilidade	21	100	21	1	5	0	-
	7.	Incluir um(a) revisor(a)	21	100	21	0	-	0	-
	8.	Ler e analisar título e resumo (4 resumos)	20	95	20	3	15	16	80
	9.	Decidir sobre a pertinência do estudo com base em critérios de elegibilidade.	21	100	21	0	-	0	-
	10	Decidir sobre a elegibilidade do estudo	21	100	21	0	-	0	-
	11.	Incluir comentários, se necessário ^†^	21	100	21	0	-	0	-
3	12.	Comparar as decisões sobre a elegibilidade de cada estudo	19	90	19	13	68	5	26
	13	Resolver as divergências entre revisores(as)	19	90	19	1	5	0	-
	14	Utilizar um filtro para identificar os resumos elegíveis ^†^	12	57	12	1	8	0	-
	15	Abrir um arquivo em PDF ^‡^ com resumos elegíveis ^†^	19	90	19	0	-	0	-
4	16.	Ler a notificação da revisão em que foi convidado	21	100	21	3	15	11	52
	17.	Selecionar a revisão em que foi convidado	20	95	20	10	55	0	-
	18.	Ler e analisar título e resumo	20	95	20	1	5	8	40
	19.	Decidir sobre a pertinência do estudo com base em critérios de elegibilidade	20	95	20	0	-	0	-
	20.	Decidir sobre elegibilidade do estudo	20	95	20	0	-	0	-
	21.	Incluir comentários, se necessário ^†^	15	71	15	0	-	0	-
**Total**			**411**	**93**	**411**	**34**	**9**	**46**	**11**

* Etapa: 1) Eliminar duplicatas; 2) Identificar estudos elegíveis; 3) Resolver divergências entre revisores; 4) Identificar estudos elegíveis como revisor convidado

^†^ Tarefas não obrigatórias para conclusão da etapa de maneira completa e correta

^‡^ PDF = *Portable Document Format*

 Durante a resolução, emergiram erros no uso para as tarefas que foram concluídas ( Tabela 1 ) e não concluídas, como a 4 (n=4), 8 (n=1), 12 (n=1) e 13 (n=1). Essas ocorrências permitiram identificar os ajustes necessários no AReS, dependendo do tipo de erro e da consequência da sua ocorrência, podendo (ou não) interferir em sua eficácia ou eficiência. O detalhamento dos erros em uso observados e o resultado da análise são apresentados na Figura 2 . 

**Figura 2:** - Ações realizadas pelos participantes que interferiram nas propriedades de qualidade do sistema “Apoio à Revisão Sistemática” ou problemas na interface do sistema computacional que não permitiram o resultado desejado

### Eficiência

 A utilização do AReS requereu um tempo total médio de 55,1 minutos (desvio-padrão=15,8). Para resolução das tarefas, segundo objetivo pretendido em cada etapa, o tempo médio variou de 12,4 a 15,8 minutos ( Tabela 2 ). 

**Tabela 2:** - Tempo médio (minutos) para conclusão das etapas utilizando o sistema “Apoio à Revisão Sistemática” (n = 21). São Paulo, SP, Brasil, 2022

	**Etapa/objetivo**	**Tempo médio** **(minutos)**	DP*	**Mediana**	**Mínimo** **máximo**	**IC** ^†^ **95%**
1	Exclusão dos registros duplicados	12,8	7,0	11,7	4-31	10-16
2	Identificação de estudos elegíveis	15,8	6,8	14,9	7-32	13-19
3	Resolução de divergências	14,0	8,1	12,8	1-32	10-18
4	Seleção de estudos elegíveis, como convidado	12,4	4,2	12,9	4-20	11-14
Total do processo avaliativo		55,1	15,8	55,1		

* DP = Desvio-padrão

^†^ IC95% = Intervalo de confiança de 95%

### Usabilidade do AReS

 A nota final média de usabilidade do AReS resultou em 82,4; mediana 85; valores mínimo e máximo 35 e 97,5 (em escala de 0 a 100). A classificação do resultado desse construto por participante foi: “melhor imaginável” (n=12; 57%), “excelente” (n=5; 24%), “bom” (n=3; 14%) e “pobre” (n=1; 5%). Na Figura 3 , são apresentadas as frequências, as notas finais médias e IC95%, segundo as categorias de usabilidade no uso do sistema computacional. Observa-se que não foram apresentados a média e o IC95% para a categoria “pobre”, uma vez que tem um participante. Por meio do IC95%, pode-se afirmar que a categoria “melhor imaginável” se destaca quando comparada às categorias “bom” e “excelente”, sendo esta última a categoria de maior representação de participantes ( Figura 3 ). 

**Figura 3:** - Distribuição de participantes, segundo escore (A) e nota média por categoria de usabilidade (B) do instrumento *System Usability Scale* para o sistema “Apoio à Revisão Sistemática”

*IC95% = Intervalo de confiança de 95%

#### Condições gerais observadas durante o teste de uso do AReS

 O sistema operacional mais utilizado foi o *Microsoft Windows* e navegador *Chrome* (n=18), seguido de *MacOS* com navegador *Safari* (n=2). Foram observados erros no *Safari* , como não importação de arquivos e seleção automática de decisão sobre a elegibilidade dos estudos, mostrando a necessidade de ajustes no AReS. Durante o teste, os participantes passaram a utilizar o navegador *Chrome* para melhor uso do sistema computacional e para completar as tarefas 3 e 8. 

A câmera foi mantida aberta pela maioria dos participantes (n=14). Um deles disse que a manteria fechada devido a problemas em seu funcionamento. Foi solicitado ao participante que “pensasse em voz alta”, e nove (43%) seguiram essa instrução. A maioria dos participantes compartilhou a tela inteira, entretanto, quando era compartilhada apenas a aba de uso do AReS, a pesquisadora não podia verificar o tipo de material explicativo utilizado.

 A remarcação do teste foi necessária para dois participantes por motivos de lentidão na internet e problemas em seu computador. O sistema AReS apresentou problemas ao se pressionar o ícone para selecionar a revisão em que o participante foi convidado (Tarefa 17), sendo necessário pressionar o ícone “atualizar” ou fazer novo *login* . Na atividade 2 (revisor convidado), o sistema computacional apresentou lentidão e problemas técnicos que impediram alguns participantes de concluírem a Tarefa 21. 

Alguns participantes fizeram sugestões: usar resumos em língua portuguesa no próximo teste (n=3) e deixar claro, no início, que a participação seria como revisor principal e depois como convidado (n=1). Um participante verbalizou uma queixa sobre o grande número de resumos que deveriam ser lidos (n=8 resumos) e outro verbalizou que o PDF com as tarefas não explicava como resolvê-las, mesmo após exposição da pesquisadora de que o participante deveria ler a tarefa e tentar resolvê-la sozinho no sistema computacional.

#### Comentários dos participantes

As sugestões dos participantes sobre o AReS são apresentadas a seguir:

Melhorar a caixa de importação das bases de dados (...) para indução de seleção de arquivo de importação (P1).

Incluir um ícone de orientação sobre a atenção aos detalhes da tela e explicação de cada item (P2).

Achei o sistema bem intuitivo. Porém, não identifiquei como fazer a mudança de elegibilidade quando há divergência entre os autores. O gráfico que fica no dashboard me mostrou apenas a porcentagem de trabalhos elegíveis para cada autor, dando uma falsa impressão de não haver divergências. Mas, ao olhar novamente, havia divergências (P3).

Acredito que a interface com o usuário é difícil e pouco intuitiva, é difícil descobrir o próximo passo. Acredito que falta caixas informativas sobre como operar o sistema ou como realizar determinada tarefa. Não sei como seria se tivesse um treinamento prévio sobre o sistema, como seria a capacidade de uso, mas, como os testes foram sem um treinamento completo (somente alguns dados de apostila), senti bastante dificuldade (P4).

### Ajustes e melhorias

 Entre os ajustes e melhorias, incluiu-se: padronizar, em todos os navegadores, o texto da caixa de seleção de arquivos para *upload* ; incluir balões explicativos sobre os botões das telas do sistema computacional; incluir botão para quebra de cegamento, para facilitar a exibição da comparação das respostas dos revisores; permitir a seleção de uma revisão pelo menu “Revisões” (e não apenas pela barra superior); listar, separadamente, as revisões criadas pelo próprio pesquisador e as revisões em que foi convidado a participar; na tela de resumos, exibir o botão “salvar” apenas quando houver inclusão de um comentário; permitir clicar no título do estudo para acessar o resumo; na identificação de duplicatas, retirar a opção de selecionar grupo para comparação das referências. 

## Discussão

 O presente estudo avaliou o sistema AReS, que apresenta funcionalidades para as fases iniciais de estudos de revisão de literatura, estruturado de acordo com metodologia criteriosa ^(^ 1 ^,^ 3 ^)^ , seguindo também os requisitos de normativa internacional ^(^ 16 ^)^ . A avaliação empírica apresentou alto grau de usabilidade percebida por parte dos avaliadores voluntários, os quais eram estudantes de pós-graduação e que constituíram, não intencionalmente, um grupo heterogêneo quanto ao uso de ferramentas computacionais em estudos de revisão, sendo representativos do público-alvo. 

 Deve-se sempre considerar a possibilidade de viés de seleção ao se utilizar amostra por voluntários, uma vez que a motivação pelo *software* pode ter sido pelo interesse em conhecer a nova ferramenta ou pela oportunidade de aprender sobre seu uso ^(^ 22 ^)^ . A segunda opção é corroborada pela porcentagem de 67% de participantes que relataram a não utilização anterior desse tipo de ferramenta. Tal característica pode ter provocado um efeito positivo no estudo, uma vez que poderiam necessitar de mais recursos computacionais e exigido mais do sistema em avaliação ^(^ 23 ^)^ . 

A opção metodológica pelo não treinamento prévio permitiu a exposição a um novo produto e favoreceu o surgimento de informações relativas às dificuldades de uso devido às interfaces do sistema computacional, conferindo ao teste genuinidade e cumprimento do objetivo do estudo.

 As tarefas propostas no teste em uso foram criadas pensando-se em um passo a passo ^(^ 24 ^)^ para a realização de uma revisão, seguindo uma sequência em quatro etapas, que favorecia a verificação da eficácia, eficiência e o monitoramento dos problemas de interface. Como a qualidade de uma revisão depende de seleção não enviesada de estudos ^(^ 3 ^)^ , a estratégia de mais de um avaliador foi contemplada no AReS, resultando em uma avaliação mais longa, feita em duas fases, cada uma com papéis diferentes para o revisor (principal e convidado). 

Os participantes alcançaram os objetivos das etapas em altos percentuais, com taxa de conclusão de pelo menos 90%, sendo consideradas satisfatórias pelos autores, conferindo ao processo avaliativo a qualidade de validade interna, indicando que os recursos metodológicos utilizados foram capazes de captar a adequação das funcionalidades do AReS.

 Para as tarefas 14 (uso de filtro para identificar os resumos elegíveis) e 21 (inclusão de comentários, se o estudo for considerado não elegível pelo revisor convidado) que tiveram taxas de conclusão inferiores a 90%, a influência no desempenho do *software* é mínima, indicando por meio da tarefa 14 possível problema na especificação da tarefa, e não na interface. Com a segunda ocorrência, pode-se identificar necessidade de correção de problemas técnicos no sistema. Apenas a tarefa 12 foi considerada complexa, pois envolvia acesso a mais de um menu para alcançar a conclusão, justificando a assistência requerida pela maioria dos participantes e indicação de necessidade de inclusão de um botão de atalho. 

 O tempo médio do teste de usabilidade variou conforme o esperado pelos investigadores. Em situação de condução de uma pesquisa, o tempo médio de leitura e análise de títulos e resumos (tarefa 8) varia conforme a quantidade de referências recuperadas nas bases de dados, enquanto o tempo para resolução das divergências pode variar segundo a quantidade de resumos a serem comparados ^(^ 25 ^)^ . Os achados do presente estudo corroboram com a literatura que indica que as fases iniciais de revisões são as que requerem maior tempo de trabalho para a conclusão da seleção de estudos elegíveis, assim, a preferência é por recursos que favoreçam redução do tempo de dedicação a essa etapa ^(^ 13 ^)^ , em relação ao trabalho manual e ferramentas não específicas, como planilhas. 

 O AReS foi avaliado com grau de usabilidade positivo, com o “melhor imaginável” em 57% das avaliações; 24% como “excelente” e 14% como “bom”. O questionário SUS foi de fácil aplicação, sendo um instrumento validado e amplamente utilizado em pesquisas e em testes conduzidos por profissionais de tecnologia da informação, inclusive na área da saúde ^(^ 26 ^)^ . Ressalta-se que o instrumento SUS também permite a comparação de diferentes sistemas *web* ^(^ 27 ^)^ , com usuários em diferentes regiões geográficas ^(^ 28 ^)^ . 

 A ocorrência de erros no uso, como a não identificação de todas as duplicatas, de forma geral, indicou a necessidade de maior comunicação entre o sistema computacional e o usuário, resolvido por ajustes na interface e inclusão de mensagens explicativas. Identificar os problemas em uso e corrigi-los confere ao produto maior uso intuitivo e maior adesão do público alvo ^(^ 29 ^)^ . 

O teste realizado de maneira remota já é descrito na literatura e foi importante por permitir ao participante utilizar seus recursos tecnológicos, possibilitando o teste de usabilidade em contexto real de uso. Permitiu, ainda, a gravação da sessão de trabalho e o posterior registro de dados, como a identificação da completude, o cumprimento do objetivo da tarefa e a ocorrência de erros no uso. O uso de ferramenta para teleconferência foi fundamental e permitiu a interação entre os envolvidos, facilitou o compartilhamento de materiais instrutivos das atividades, a observação e gravação das telas, sem a necessidade de custos adicionais com impressão de materiais e equipamentos para filmagem.

 Embora existam diferenças na conectividade intramunicipal ^(^ 30 ^)^ , as condições para a condução do teste foram consideradas adequadas pelos pesquisadores. Segundo a literatura, o teste remoto síncrono não difere significantemente dos testes realizados em laboratório ^(^ 31 ^)^ e favorece a participação de pessoas localizadas em diferentes regiões do país ou do exterior, podendo ser preferido em pesquisas com baixo orçamento para esse tipo de avaliação e demandas específicas, como o isolamento social imposto pela pandemia de COVID-19. 

 Como potencialidade deste estudo, era mandatória a realização do teste de usabilidade conduzido conforme procedimentos metodológicos reconhecidos, para disponibilidade de um *software* confiável, eficaz e com alto grau de usabilidade. Tecnologias que produzam resultados acurados são cruciais para auxiliar na tomada de decisão e garantir a segurança e acurácia de dados. Incluir detalhadamente, neste trabalho, o processo avaliativo do produto pode auxiliar os profissionais de saúde que participam de outros processos avaliativos, pois apresenta ferramentas específicas para a avaliação de um *software* , fundamenta as escolhas de uso e explicita as propriedades de qualidade a serem avaliadas. 

 As características importantes para a escolha de programas computacionais específicos em revisão sistemática são a simplicidade, a facilidade de aprendizagem e a intuitividade do *layout* ^(^ 12 ^)^ . Procurou-se, na construção do produto apresentado, alcançar tais atributos. Para controlar o viés na seleção de estudos elegíveis, incorporou-se no AReS a possibilidade de participação de mais de um avaliador e o cegamento das decisões, garantindo a independência entre suas decisões. Outra característica do AReS é a apresentação, em uma só tela, do resumo do artigo e dos critérios de elegibilidade que, ao serem preenchidos, conduzem à decisão de serem elegíveis (ou não). Essa funcionalidade não foi encontrada em nenhum outro sistema, sendo uma proposta original e única do AReS 

As limitações do estudo referem-se ao motivo da construção do AReS, que teve origem na necessidade específica da pesquisadora na operacionalização de uma revisão. O sistema é apresentado somente em língua portuguesa, incluindo os materiais de apoio. O instrumento utilizado para avaliação de usabilidade (SUS) é validado para a língua portuguesa em Portugal; no planejamento do teste, não foi recuperado estudo detalhado de adaptação transcultural desse instrumento para o contexto brasileiro.

Ainda, como limitação metodológica, pode-se mencionar a não homogeneidade do procedimento de abertura das câmeras durante o teste em uso. Entretanto, tal fato parece não ter interferido nos resultados, uma vez que não era de interesse avaliar a dimensão comportamental no uso da ferramenta. O não compartilhamento da tela durante o uso de material explicativo também foi um fator limitante. Ressalta-se, no entanto, que a pesquisadora solicitava essa informação para registro em ficha. O não esclarecimento a um participante sobre o papel que iria desempenhar como revisor principal ou convidado pode ser considerado como uma falha metodológica do teste. Essa ocorrência foi verbalizada pelo segundo participante e as orientações passaram a ser dadas a todos os demais.

 Em relação a outras ferramentas computacionais, a presente versão do AReS possui funcionalidades para eliminação de duplicatas e seleção de estudos, ao contrário das que apoiam outras fases de uma revisão sistematizada, como a extração de dados, auxiliada por *DistillerSR* ^(^ 32 ^)^ . O AReS não destaca palavras-chave, nem permite a importação de artigos completos, como o *Rayyan* . 

 Como visão de futuro, pode-se destacar que o desenvolvimento de funcionalidades para outras etapas de revisão, utilizando aplicações de inteligência artificial, assim como outras ferramentas computacionais disponíveis ^(^ 33 ^-^ 34 ^)^ , poderá ampliar seu uso por estudantes, pesquisadores e profissionais da área da saúde. 

## Conclusão

 O AReS apresenta parâmetros de eficácia e eficiência necessários a uma ferramenta computacional de revisão. Apresenta usabilidade de 82,4 em escala de 0 a 100 avaliada pelo instrumento *System Usability Scale* (SUS). A versão apresentada incorpora os ajustes identificados no teste em uso. O AReS é uma ferramenta útil e de fácil utilização, voltada ao meio acadêmico e de pesquisa, proposta para diminuir o tempo de trabalho em fases iniciais de revisões de literatura.
